# Intracellular pathogen *Leishmania* intervenes in iron loading into ferritin by cleaving chaperones in host macrophages as an iron acquisition strategy

**DOI:** 10.1016/j.jbc.2022.102646

**Published:** 2022-10-26

**Authors:** Sandhya Sen, Saswat Kumar Bal, Sameeksha Yadav, Pragya Mishra, Vishnu Vivek G, Ruchir Rastogi, Chinmay K. Mukhopadhyay

**Affiliations:** Special Centre for Molecular Medicine, Jawaharlal Nehru University, New Delhi, India

**Keywords:** ferritin, iron, chaperone, *Leishmania*, macrophage, host–pathogen interaction, metalloprotease, cDNA, complementary DNA, CS, culture supernatant, FBS, fetal bovine serum, Fe, iron, Ft, ferritin, HIFα, hypoxia-inducible factor alpha, hnRNP, heterogeneous nuclear ribonucleoprotein, IgG, immunoglobulin G, IP, immunoprecipitation, J774, J774A.1, KH, K-homology, LD, *Leishmania donovani*, LIP, labile iron pool, MOI, multiplicity of infection, NTA, nitrilotriacetic acid, *o*-phe, *o*-phenanthroline, PCBP, poly(rC)-binding protein, PHD, prolyl hydroxylase, poly-C, polycytosine, SLA, soluble leishmanial antigen, TPEN, *N*,*N*,*N*′,*N*′-tetrakis (2-pyridinylmethyl)-1,2-ethylenediamine

## Abstract

Iron (Fe) sequestration is one of the most important strategies of the host to control the growth and survival of invading pathogens. Ferritin (Ft) plays a pivotal role in the sequestration mechanism of mammalian hosts by storing Fe. Recent evidence suggests that poly(rC)-binding proteins (PCBPs) act as chaperones for loading Fe into Ft. Incidentally, modulation of host PCBPs in respect to storing Fe in Ft during any infection remains unexplored. Among PCBPs, PCBP1 and PCBP2 are present in every cell type and involved in interacting with Ft for Fe loading. *Leishmania donovani* (LD) resides within macrophages during the mammalian stage of infection, causing life-threatening visceral leishmaniasis. Here, we reveal the ability of LD to cleave PCBP1 and PCBP2 in host monocytes/macrophages. LD cleaves PCBP1-FLAG into two fragments and PCBP2-FLAG into multiple fragments, thus affecting their interactions with Ft and resulting in decreased Fe loading into Ft. LD-derived culture supernatant or exosome-enriched fractions are also able to cleave PCBPs, suggesting involvement of a secreted protease of the parasite. Using an immune-depletion experiment and transgenic mutants, we confirmed the involvement of zinc-metalloprotease GP63 in cleaving PCBPs. We further revealed that by cleaving host PCBPs, *Leishmania* could inhibit Fe loading into Ft to accumulate available Fe for higher intracellular growth. This is the first report of a novel strategy adopted by a mammalian pathogen to interfere with Fe sequestration into Ft by cleaving chaperones for its survival advantage within the host.

Iron (Fe) plays a critical role in the host–pathogen interaction. Pathogens must acquire Fe from the host for survival, growth, and virulence (1). So they subvert existing mechanisms of host Fe homeostasis toward their benefits. In contrast, hosts try to block Fe availability to invading pathogens by employing sequestration mechanisms (1, 2). Storing Fe into ferritin (Ft) in mammalian hosts is one of the most recognized mechanisms of Fe sequestration during infection (3, 4). Ft consists of two subtypes (H and L) of 24 units. It may store up to 4500 Fe atoms (5). It needs an effective Fe chaperone system to load Fe into Ft. Recent evidences established the role of poly(rC)-binding proteins (PCBPs) as Fe chaperones for Ft (6).

PCBPs are multifunctional proteins that bind to specific polycytosine (poly-C) stretch in DNA and RNA with high affinity to regulate gene expression at transcriptional and post-transcriptional levels. They belong to the heterogeneous nuclear ribonucleoprotein (hnRNP) family. Five members of hnRNP family have been identified so far in mammalian cells. Those are PCBP1, PCBP2, PCBP3, PCBP4, and hnRNP K. Among them, four isoforms of PCBPs are recently recognized as cytosolic Fe chaperones (6). PCBP1 and PCBP2 are abundantly and ubiquitously expressed in all the mammalian tissues (7, 8, 9). They bind Fe and form complex with cellular Ft for loading Fe into later (6, 10). PCBP3 and PCBP4 are minimally expressed in some tissues and only at some phases of development (11). In fact, little is known about their cellular functions, although both have been demonstrated to bind poly-C stretch of oligonucleotides (6). PCBP1 and PCBP2 are closely related proteins and 83% identical to each other (12). PCBP1 is encoded by an intronless gene that is believed to be generated by the retrotransposition of PCBP2 mRNA, whereas several forms of mRNAs are generated from the genes of other PCBPs because of alternative splicing. All the members of PCBP family consist of three highly conserved hnRNP K-homology (KH) domains that not only bind to poly-C stretch of nucleic acids with high affinity but also mediate their interactions within the RNP complex. PCBP1 and PCBP2 consist of two nuclear localization signals (8, 13), one between the KHII and KHIII domain and the other one is mapped within the KHIII domain (14). It is also reported that PCBP1 and PCBP2 are modified during viral infections and are hijacked for the replication of the respective viral genomes (15). Furthermore, PCBPs were also found to bind to the internal ribosome entry site of some viral RNAs to serve as translational coactivators and to mediate cap-independent translation (16, 17). However, the impact of any infection on the fate of PCBPs with regard to their Fe chaperon activities is yet to be reported.

Leishmaniasis is a neglected tropical disease. An estimated 350 million people are at risk of contracting the disease in 88 countries around the globe. Visceral leishmaniasis, caused by *Leishmania donovani* (LD) or *Leishmania infantum*, is the most severe form of the disease, and if left untreated, 95% of cases are fatal (18). *Leishmania* is an obligate parasite that shuttles between sand fly vector and vertebrate host. It transforms into motile and infectious metacyclic promastigote in the gut lumen of sand fly. In the vertebrate host, the *Leishmania* promastigote differentiates into an obligatory intracellular amastigote within macrophages (19). The intracellular parasites need to acquire Fe to maintain optimal metabolic activities and for their self-defense (20) like acting as a cofactor in antioxidant enzyme Fe superoxide dismutase (21, 22). The supplementation of Fe in *Leishmania*-infected hamsters was reported to promote the multiplication of the parasites (23) implicating dependence of *Leishmania* on the availability of host Fe for its growth and survival within the hostile microenvironment of the macrophages (24, 25).

We reported earlier that LD could use host labile iron pool (LIP) for its intracellular growth (24). In response, the Fe storage capacity of the host should be increased to deny Fe availability to the parasite. LD infection results in increased Fe uptake (24) and decreased Fe release component of the host macrophage (25). Since Ft expression and its Fe-storing capacity depend on LIP, the maintenance of normal Fe loading into host Ft may be disadvantageous to the intracellular *Leishmania*. Thus, it needs a strategy to overcome the challenge of Fe sequestration into Ft by the host. In this study, we reveal that LD cleaves PCBP1 and PCBP2 to affect their interactions with Ft in host macrophages resulting in decreased Fe loading into Ft. This allows the increased availability of host Fe pool for the intracellular *Leishmania*. Evidences suggest the involvement of zinc (Zn)-containing metalloprotease GP63 in cleaving PCBP1 and PCBP2. The impact of any infection on host Fe chaperones and resultant compromised Fe sequestration into Ft has not been reported so far.

## Results

### LD affects PCBP1 and PCBP2 protein levels in host macrophages

**Figure 1:**
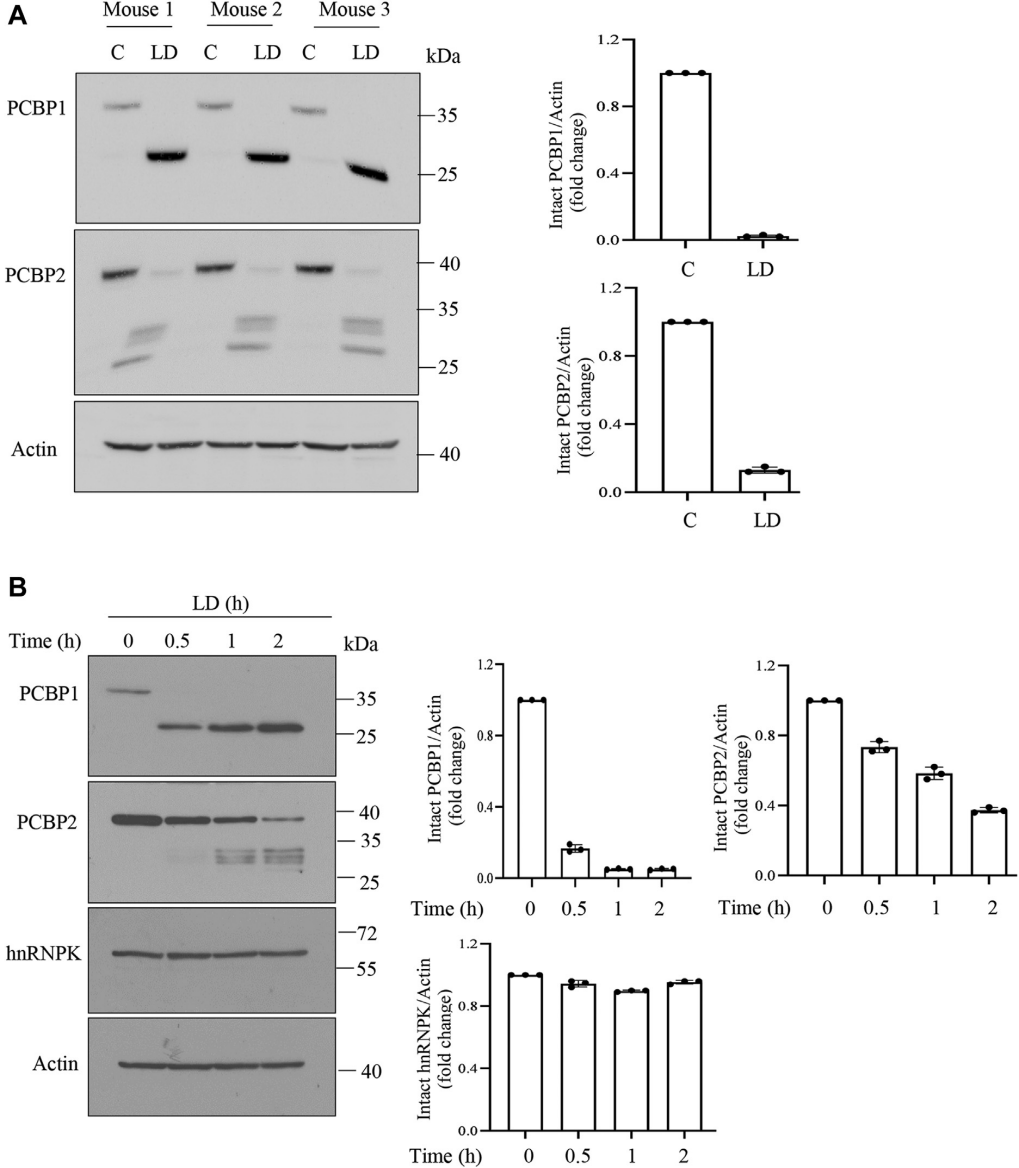

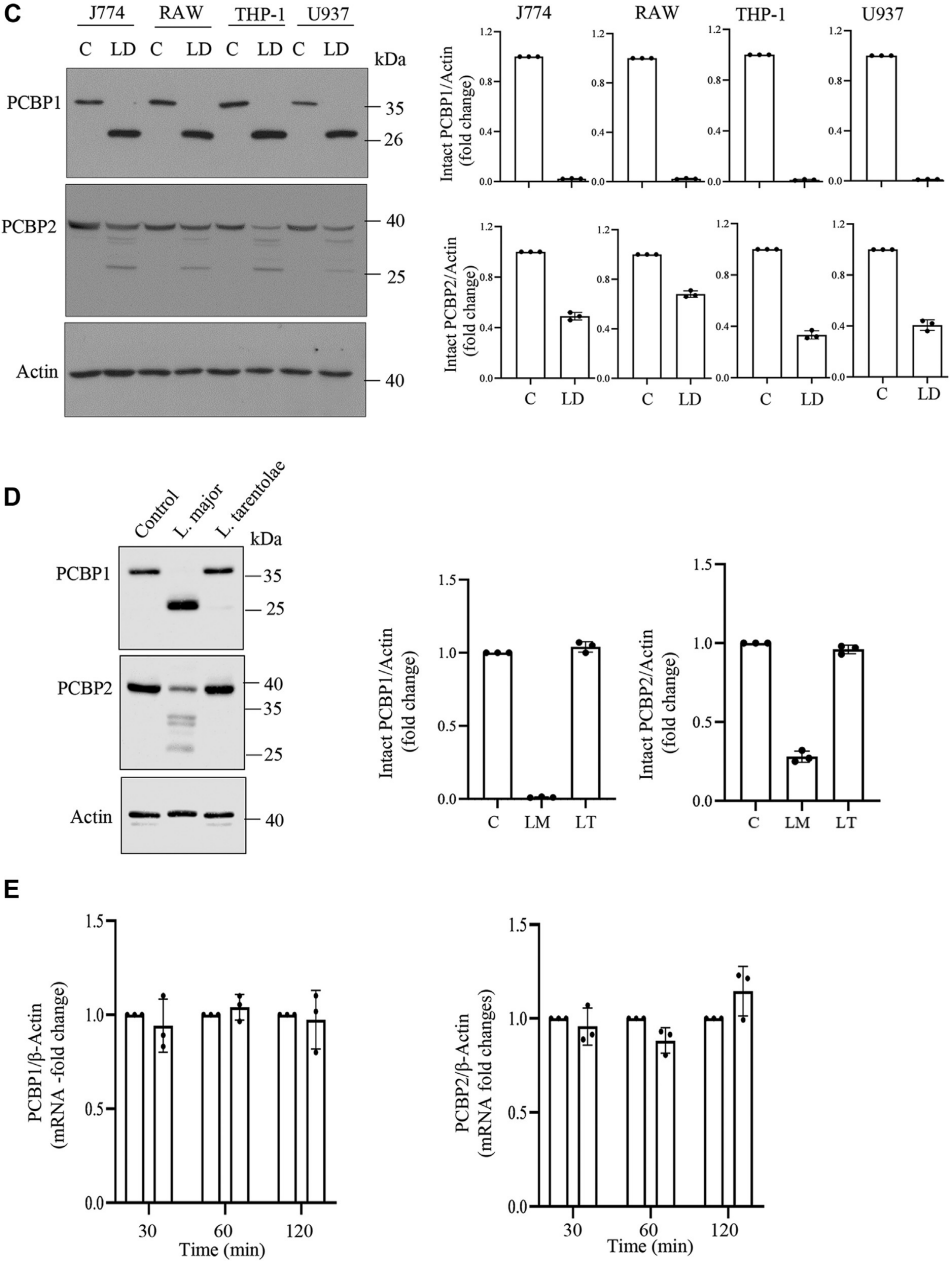
***Leishmania* infection affects iron chaperon PCBP1 and PCBP2 protein expression in host macrophages.***A*, splenocytes were isolated from mouse (n = 3) and subjected to *Leishmania donovani* (LD) infection for 2 h (MOI = 1:10) or kept uninfected. Cell lysates were immunoblotted using PCBP1, PCBP2, and actin antibody. *B*, J774 macrophages were infected with LD (MOI = 1:10) for 0 to 2 h. Cell lysates were immunoblotted using PCBP1, PCBP2, hnRNP K, and actin antibody. *C*, murine macrophage cell lines (J774 and RAW264.1) and human monocytic cell lines (THP1 and U937) were infected with LD for 2 h (MOI = 1:10) or kept uninfected and subjected to immunoblot analyses using PCBP1, PCBP2, and actin antibodies. *D*, J774 cells were infected with *Leishmania major* (LM) and *Leishmania tarentolae* (LT) (MOI = 1:10) for 4 h, and immunoblot was performed using PCBP1, PCBP2, and actin antibody. Results are representatives of one of the three independent experiments. *Right panels* show quantifications of intact PCBP1 or PCBP2 representing ±SD from three independent experiments. *E*, mice splenocytes were isolated and infected with LD (up to 120 min). PCBP1 and PCBP2 mRNA expressions were determined from total RNA using quantitative RT–PCR. β-Actin was used as endogenous control. Data represent mean ± SD from three independent experiments performed in triplicates. hnRNP, heterogeneous nuclear ribonucleoprotein; J774, J774A.1; MOI, multiplicity of infection; PCBP, poly(rC)-binding protein.

Among PCBPs, PCBP1 and PCBP2 are primarily involved in Fe loading into Ft (6). So, we examined protein levels of PCBP1 and PCBP2 in infected host macrophages. Splenocytes isolated from three mice were infected with LD and analyzed by Western blot. Results showed a strong decrease in protein abundance of both PCBP1 and PCBP2 with simultaneous appearance(s) of faster migrating band(s) in infected cells (Fig. 1*A*). J774A.1 (J774) macrophage is a well-established cellular model to understand *Leishmania*-induced alterations in host cells (24, 25, 26). Intact PCBP1 band became almost undetectable within 30 min of infection in J774 cells, whereas intact PCBP2 level was decreased steadily with increasing time of infection as detected in Western blots (Fig. 1*B*). There was no effect on another ribonucleoprotein hnRNP K (Fig. 1*B*). We detected a faster migrating band of higher intensity of PCBP1 at about 26 kDa in infected J774 cells and splenocytes, whereas multiple fragments between 35 and 25 kDa were detected for PCBP2 (Fig. 1, *A* and *B*). LD infection (2 h) in other phagocytic cell lines like murine macrophage RAW264.1, human monocytic THP1, and U937 also resulted in decreased PCBP1 and PCBP2 protein levels with appearance of faster migrating bands (Fig. 1*C*). We also detected decreasing trend of PCBP1 and PCBP2 protein levels with increase in multiplicity of infection (MOI = 1:1, 1:5, 1:10, and 1:20; 2 h) and simultaneous appearances of faster migratory band(s) (Fig. S1). Interestingly, Western blot analysis always showed a more intense faster migrating band in infected cells than the intact PCBP1 in uninfected cells. The reason behind the higher intensity of the faster migrating band is not clear. We detected a similar effect on host PCBP1 and PCBP2 protein by *Leishmania major* but not by *Leishmania tarentolae* infection (Fig. 1*D*). Investigation on the regulation of PCBP1 and PCBP2 transcripts after the infection (0–2 h) showed no significant alteration by quantitative PCR analysis in splenocytes (Fig. 1*E*) and by semiquantitative RT–PCR in J774 cells (0–8 h) (Fig. S2).

### LD promotes cleavage of PCBP1 and PCBP2 in host macrophages

**Figure 2:**
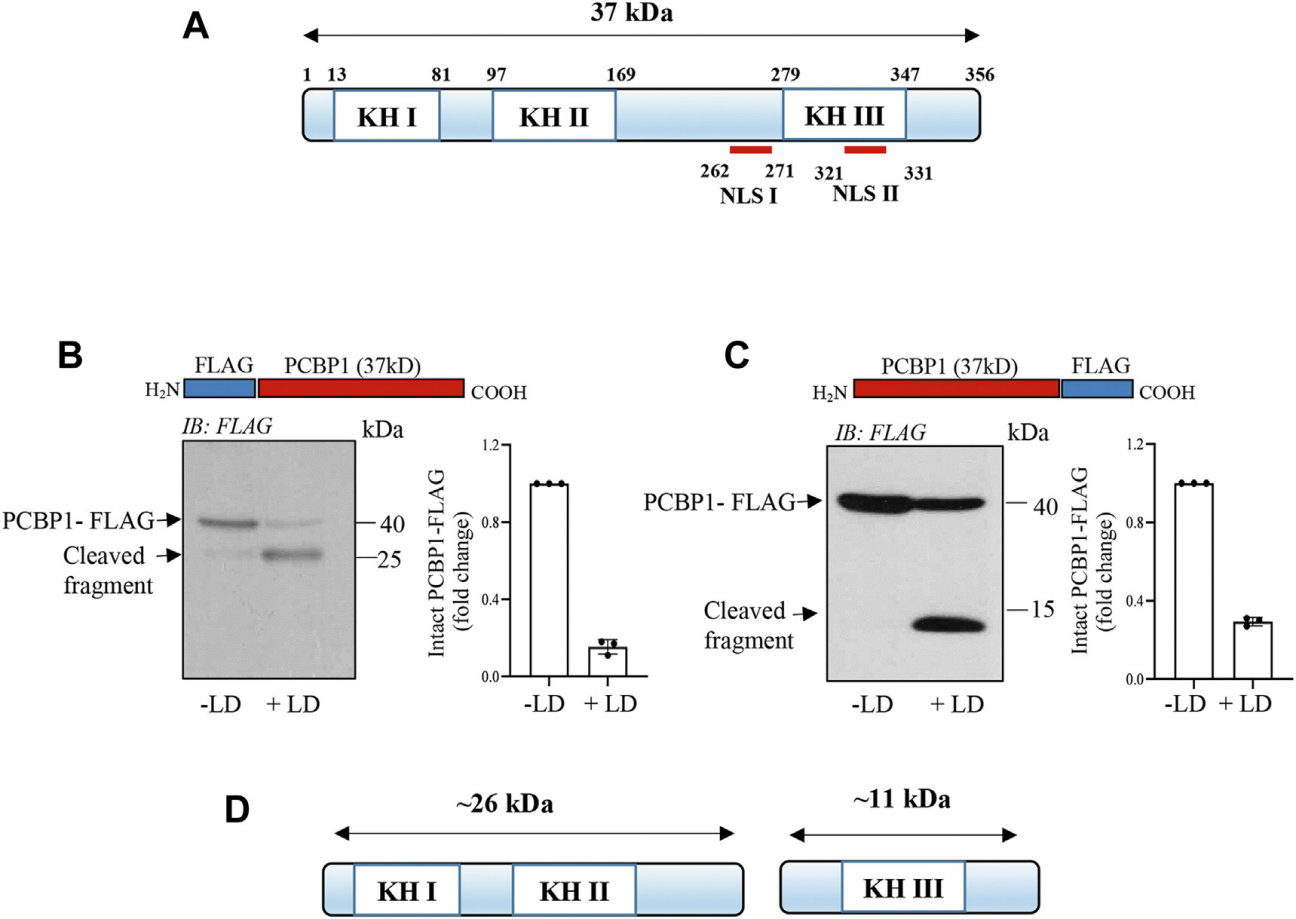
**Cleavage of PCBP1 by *Leishmania donovani* (LD) infection.***A*, a schematic representation of mouse PCBP1 indicating the location of three KH domains. *B*, p3xFLAG-CMV-7.1-PCBP1 cDNA was transiently transfected in J774 macrophages and then infected for 2 h with LD. Cell lysates from uninfected control and infected J774 cells were prepared and subjected to immunoblot analysis using FLAG antibody. Schematic representation of the N-terminal FLAG-tagged PCBP1 is shown on the *top of the figure*. *C*, p3xFLAG-CMV-14-PCBP1 cDNA was transiently transfected in J774 macrophages and infected for 2 h with LD. Cell lysates from uninfected control and infected J774 cells were examined by immunoblot analysis using FLAG antibody. The schematic representation of the C-terminal FLAG-tagged PCBP1 is shown on the *top of the figure*. *D*, a schematic diagram to show tentative sizes of the LD-induced cleaved products of PCBP1. Results are representatives of one of the three independent experiments. *Right panels* (*B* and *C*) represent quantitation from three independent experiments. cDNA, complementary DNA; J774, J774A.1; KH, K-homology; PCBP, poly(rC)-binding protein.

**Figure 3:**
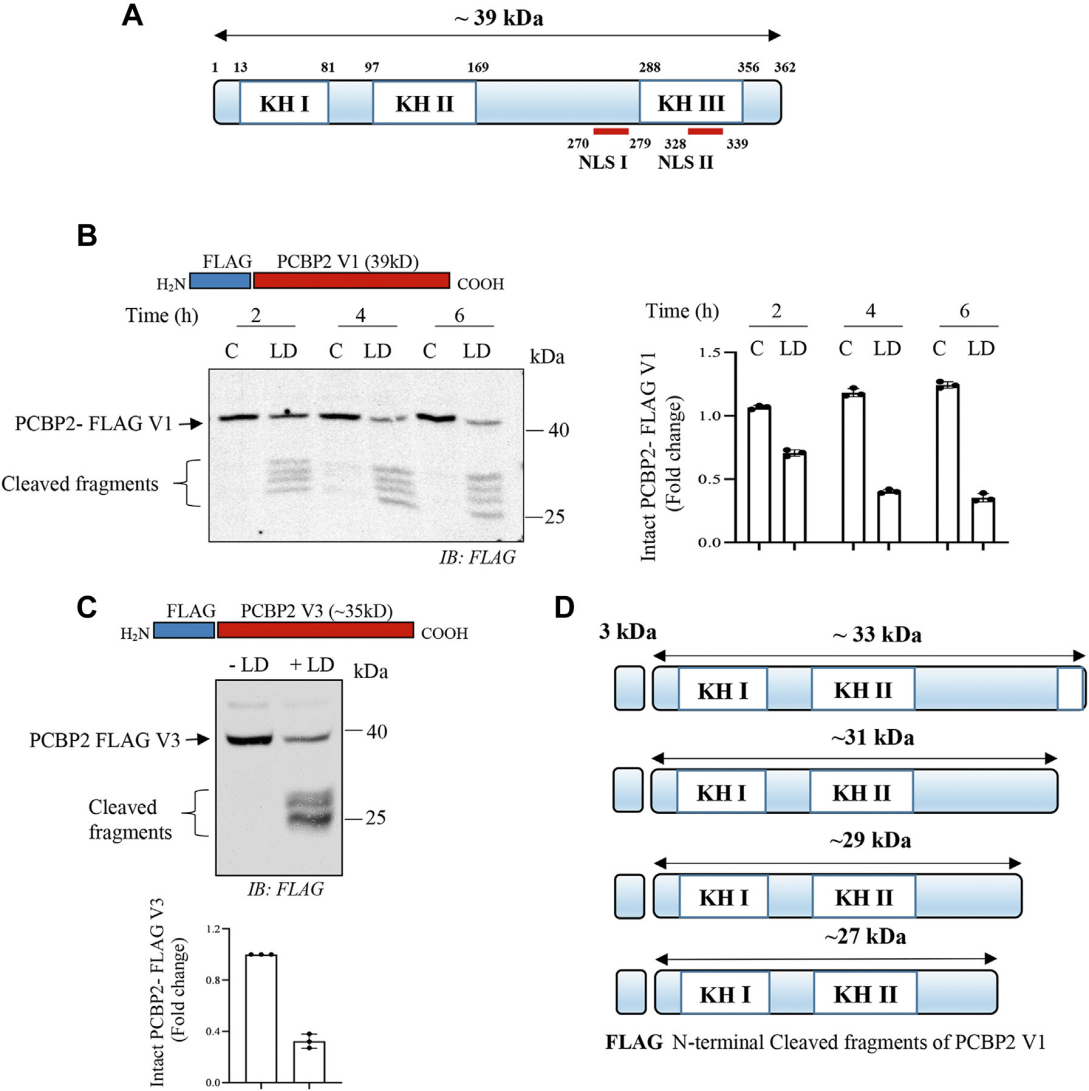
**Cleavage of PCBP2 by *Leishmania donovani* (LD) infection.***A*, a schematic representation of mouse PCBP2 indicating the location of three KH domains. *B*, J774 cells were transiently transfected with p3xFLAG-CMV-7.1-PCBP2 variant 1 cDNA and infected with LD for different periods (0–6 h). Whole cell lysates were subjected to immunoblot analysis using FLAG antibody. The schematic representation of the N-terminal FLAG-tagged PCBP2 is shown on the *top of the figure*. Quantification of intact PCBP1-FLAG V1 has been shown in the *right panel*; ±SD from three independent experiments. *C*, J774 cells were transiently transfected with p3xFLAG-CMV-7.1-PCBP2 variant 3 cDNA, and the effect of LD infection (MOI = 1:10; 4 h) was determined by Western blot analysis using FLAG antibody. Quantification of intact PCBP1-FLAG V3 has been shown in the *lower panel*; ±SD from three independent experiments. *D*, a schematic diagram shows tentative sizes of the LD-induced cleaved products of N-terminal FLAG-PCBP2 variant 1 fragment. Results are representatives of one of the three independent experiments. cDNA, complementary DNA; J774, J774A.1; KH, K-homology; PCBP, poly(rC)-binding protein.

**Figure 4:**
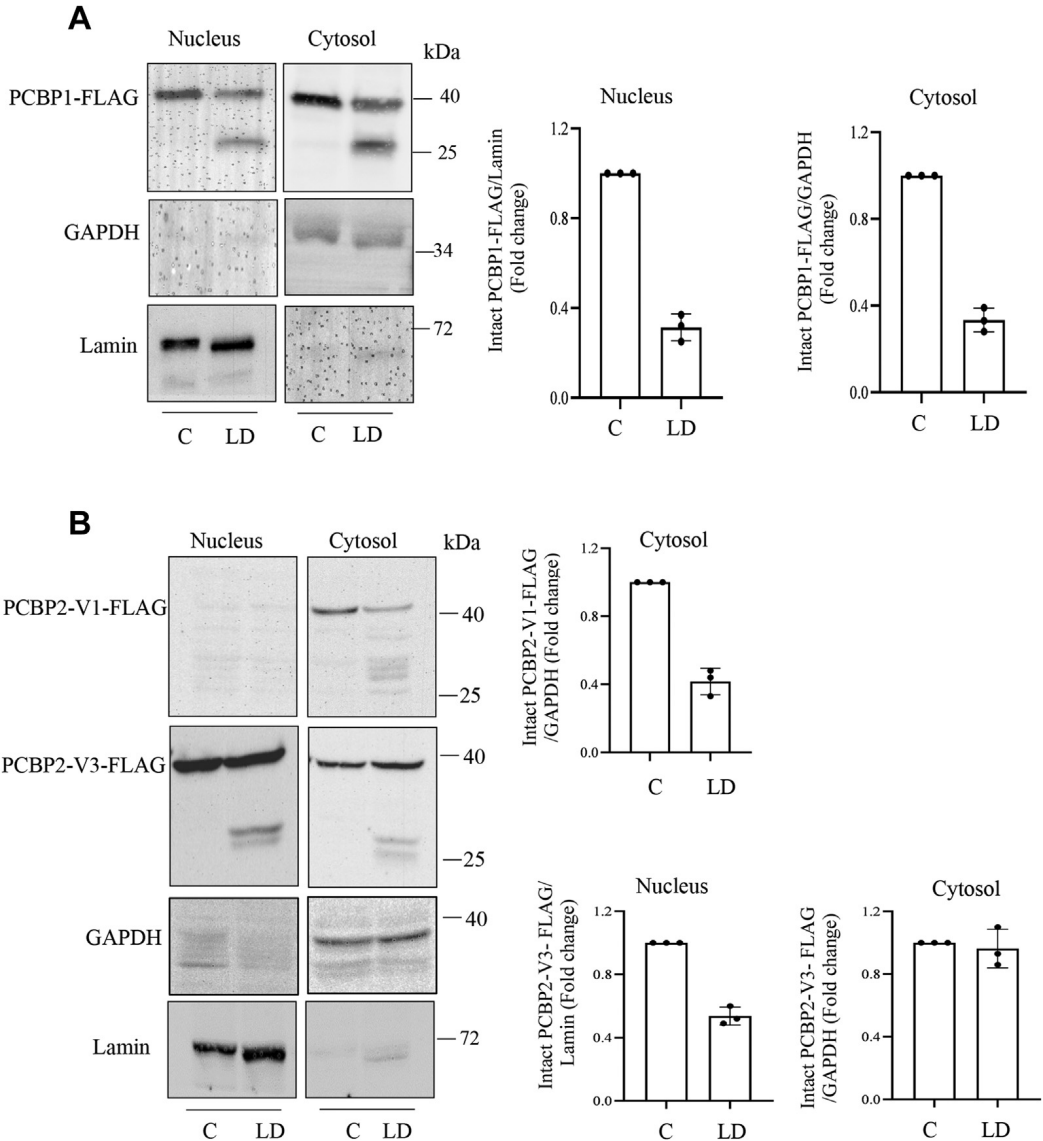
**Effect of *Leishmania donovani* infection on nuclear and cytosolic PCBP1 and PCBP2.** p3xFLAG-CMV-7.1-PCBP1 cDNA (*A*), p3xFLAG-CMV-7.1-PCBP2 variant 1 cDNA, and p3xFLAG-CMV-7.1-PCBP2 variant 3 cDNA (*B*) were transiently transfected and infected for 2 h with the parasite. Nuclear and cytosolic fractions were isolated and immunoblotted with FLAG, lamin, or GAPDH antibodies. Data shown are representatives of one of the three independent experiments. Quantification of intact PCBP1-FLAG and PCBP2 V1/V3-FLAG has been shown in *right panels*; ±SD from three independent experiments. cDNA, complementary DNA; PCBP, poly(rC)-binding protein.

Since PCBP1 abundance was decreased with simultaneous appearance of a faster moving band, we assumed that PCBP1 was cleaved because of LD infection. Murine PCBP1 protein contains 356 amino acids with three KH domains (Fig. 2*A*). To examine whether PCBP1 was cleaved, murine PCBP1 complementary DNA (cDNA) was cloned into FLAG encoding p3xFLAG-CMV-7.1 (Fig. 2*B*) and p3xFLAG-CMV-14 (Fig. 2*C*) expression vectors. J774 cells were transfected with these constructs separately. The encoded PCBP1 protein should have an N-terminal FLAG and a C-terminal FLAG tag, respectively. Transfected cells were then infected with the parasite. To understand the fate of the FLAG-tagged recombinant proteins, immunoblotting was performed. Results showed the appearance of the cleaved N-terminal FLAG-tagged PCBP1 band closer to the intact band (Fig. 2*B*), whereas cleaved fragment of the C-terminal FLAG-tagged PCBP1 was much smaller than the intact band (Fig. 2*C*). These results suggested that the PCBP1 was cleaved between the KH II and KH III domains producing ∼26 kDa N-terminal fragment and ∼11 kDa C-terminal fragment (Fig. 2*D*). It is interesting to note that the infection-induced FLAG-tagged cleaved fragments did not show higher intensity like endogenous cleaved fragment. LD infection also decreased intact PCBP2 protein band with concomitant appearance of multiple faster migrating bands suggesting its multiple cleavage because of infection (Fig. 1, *A*–*D*). The presence of four variants of PCBP2 transcripts is known in mouse cells. We detected two variants in J774 cells among them. After cloning and sequencing, they were identified as variant 1 and 3. PCBP2 variant 1 cDNA (PCBP2V1; containing 362 amino acids; about 39 kDa) was cloned into p3xFLAG-CMV-7.1 vector and expressed into J774 cells to verify the effect of LD infection (Fig. 3, *A* and *B*). The recombinant fusion protein should encode with FLAG at the N terminus (Fig. 3*B*). LD infection (0–6 h) resulted in multiple cleaved products (Fig. 3*B*). Since the FLAG is tagged at the N terminus of PCBP2, the result suggests that the cleavages happen closer to the C-terminal end like PCBP1 producing fragment sizes approximately between 33 and 27 kDa (Fig. 3*D*). Similarly, FLAG-tagged mouse PCBP2 variant 3 (containing 331 amino acids; ∼35 kDa) also showed multiple cleaved products by LD infection (Fig. 3*C*). PCBPs are present at both nucleus and cytosol. So we examined whether PCBPs are cleaved in both these locations. Result showed that N-terminal FLAG-tagged PCBP1 was cleaved both at nuclear and cytosolic fractions (Fig. 4*A*). Interestingly, we observed that N-terminal FLAG-tagged PCBP2 variant 3 was cleaved both in nuclear and cytosolic fractions (Fig. 4*B*); however, the recombinant FLAG-tagged PCBP2 variant 1 was undetectable in nucleus but was cleaved in the cytosolic fraction (Fig. 4*B*). The nuclear cleavage of PCBPs suggests the involvement of a secretory protease of the parasite because it resides within phagosome in host cells.

### LD infection affects PCBP1–PCBP2 interaction with Ft

**Figure 5:**
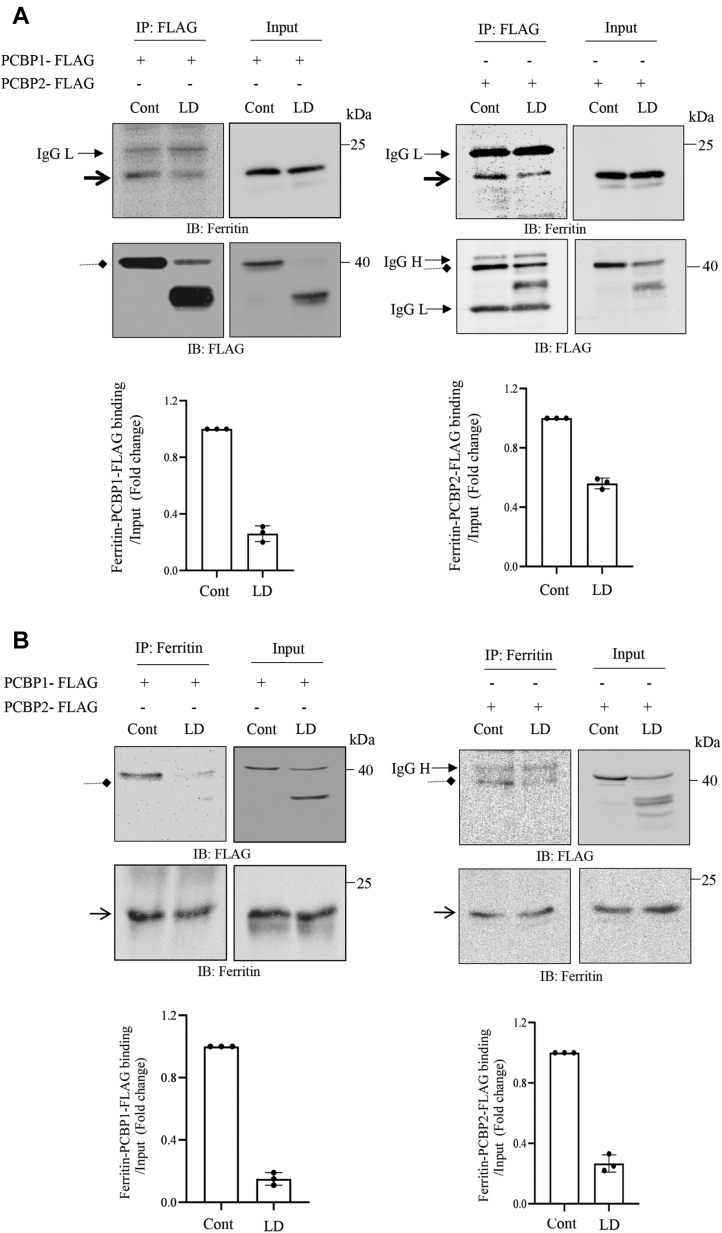
**Effect of *Leishmania donovani* (LD) infection on host ferritin (Ft) and PCBP1–PCBP2 interaction.***A*, J774 cells were transfected with either p3xFLAG-CMV-7.1-PCBP1 or p3xFLAG-CMV-7.1-PCBP2 variant 1 cDNAs and then infected with LD for 2 h. FLAG-tagged proteins were immunoprecipitated using M2 beads covalently crosslinked with A/G beads from whole cell lysates. Immune complexes were analyzed by immunoblotting using Ft or FLAG antibody as indicated in respective figures. Input was 5%. *Solid arrowheads* indicate IgG light chain (IgG L) and heavy chain (IgG H); *pointed arrowheads* indicate Ft, and *diamond arrowheads* indicate PCBP1-FLAG or PCBP2-FLAG. *B*, similarly, J774 cells were transfected with either p3xFLAG-CMV-7.1-PCBP1 or p3xFLAG-CMV-7.1-PCBP2 variant 1 and infected with *Leishmania* for 2 h. Whole cell lysates were subjected to immunoprecipitation using anti-Ft antibody covalently crosslinked with A/G beads. Then immunoprecipitated complexes were subjected to Western blot using either FLAG or Ft antibody. Input was 5%. *Solid arrowhead* indicates IgG H; *open arrowheads* indicate Ft, and *diamond arrowheads* indicate PCBP1-FLAG or PCBP2-FLAG. *Left panels* show interaction of Ft and PCBP1-FLAG, and *right panels* imply interaction of Ft and PCBP2-FLAG. Quantitation of Ft-PCBP1–PCBP2 bindings is performed from three independent experiments as ±SD and provided in *lower panels* in respective results. cDNA, complementary DNA; IgG, immunoglobulin G; J774, J774A.1; PCBP, poly(rC)-binding protein.

To examine whether cleavage of PCBPs had any impact on their interactions with Ft, J774 cells were transiently transfected with constructs encoding N-terminal FLAG-tagged PCBP1 and PCBP2V1 and then infected with the parasite for 2 h. Total cell lysates were immunoprecipitated with M2 beads (containing covalently bound FLAG antibody). After immunoprecipitation (IP), we performed immunoblot analysis with Ft antibody and detected decreased interaction of Ft with both PCBP1 (*left upper panel*) and PCBP2 (*right upper panel*) (Fig. 5*A*). We did not detect any alteration of Ft expression because of infection (Fig. 5*A*, *inputs*). When the same immunoprecipitated complexes were immunoblotted with FLAG antibody, expected cleaved products of PCBP1 (*left lower panels*) and PCBP2 (*right lower panels*) were observed. To further confirm the affected interactions between PCBPs with Ft, we also performed reverse IP after similar transfections. After IP with Ft antibody, immunoblot analysis of the complex was performed using FLAG and Ft antibody. Results showed significant decrease in interaction between PCBP1 (Fig. 5*B*; *left panel*) or PCBP2 (Fig. 5*B*; *right panel*) with Ft. There was no interaction between Ft and cleaved products suggesting the need of intact PCBPs for interaction with Ft.

### Involvement of GP63 in cleavage of PCBP1 and PCBP2

**Figure 6:**
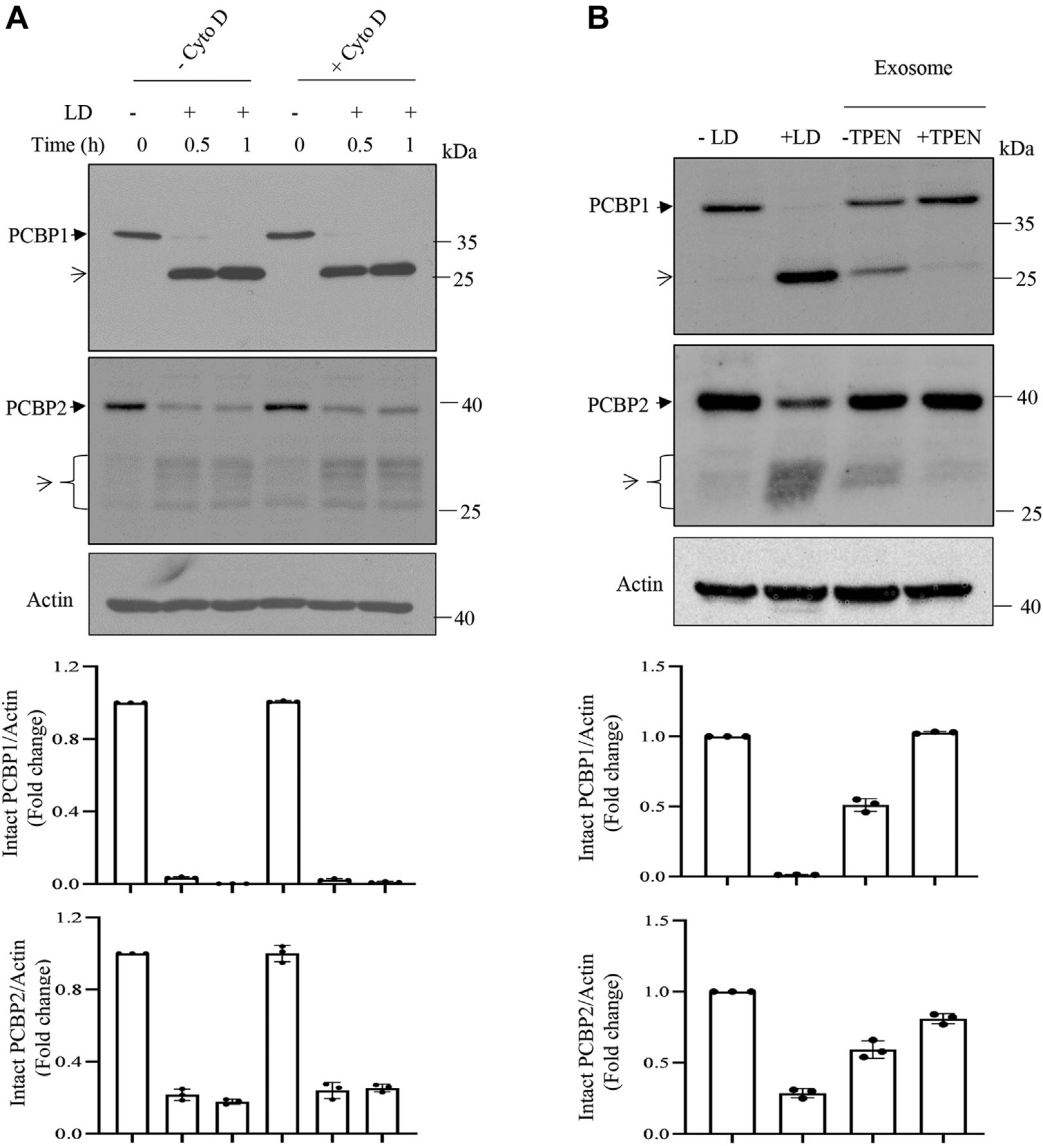

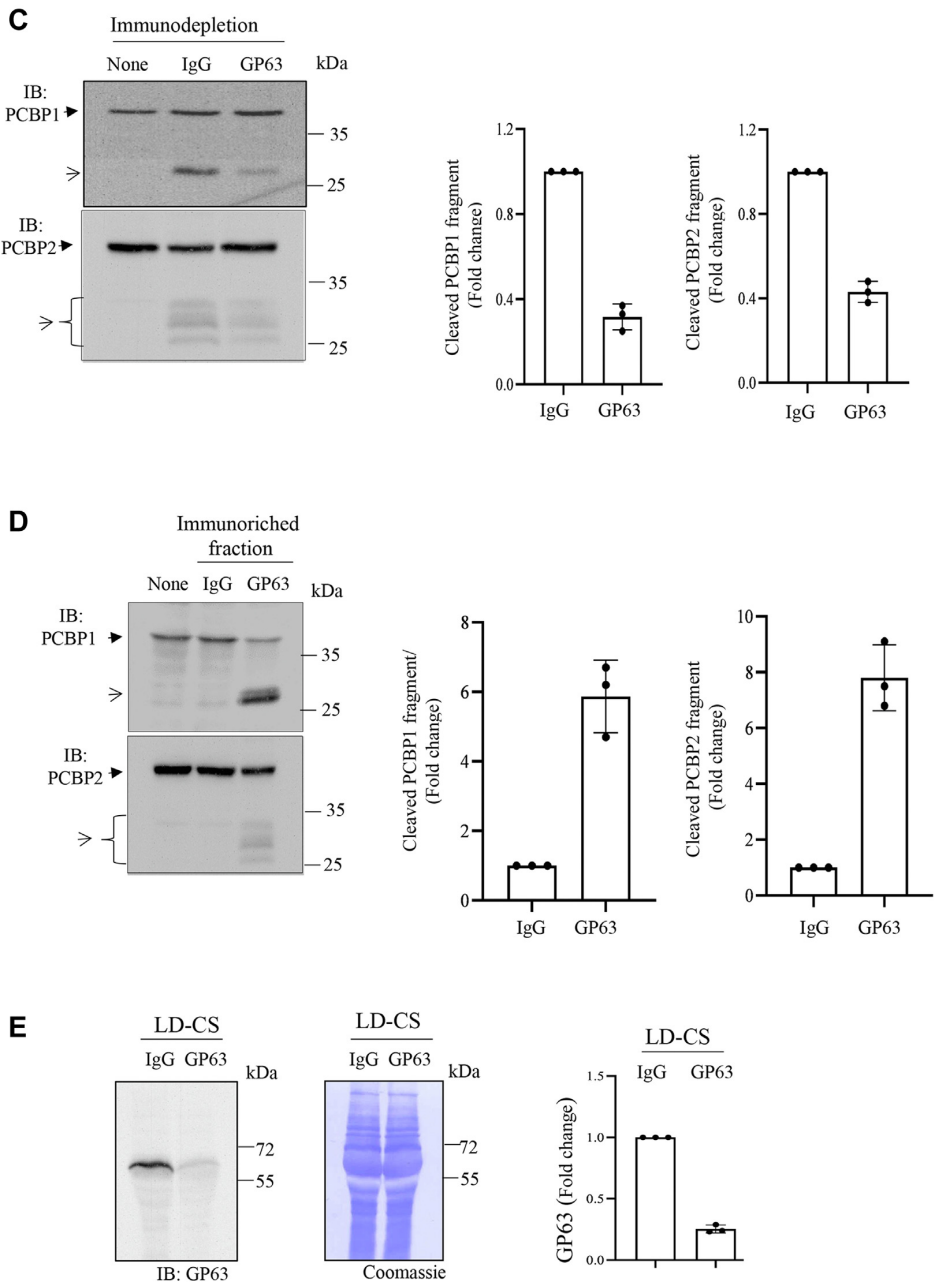
**Role of GP63 on the cleavage of PCBP1 and PCBP2.***A*, J774 cells were either treated with cytochalasin D (cyto D; 2 μM) for 1 h or kept untreated and then infected with *Leishmania donovani* (LD) for 1 h. Immunoblot analysis was performed using PCBP1 (*upper panel*) or PCBP2 (*middle panel*) antibody using total cell lysates. Actin immunoblot was done for loading control (*lower panel*). *B*, exosome-enriched fraction was isolated from LD and incubated with or without specific zinc-chelator TPEN (10 μM) for 30 min and then with J774 cells for 2 h. Total cell lysates were immunoblotted using PCBP1 (*upper panel*), PCBP2 (*middle panel*), or actin (*lower panel*) antibody. LD infection was kept as positive control. Quantitation from three independent experiments was done and shown in *lower panels* for both *A* and *B*. *Solid arrowheads* indicate intact PCBP1 or PCBP2, and *open arrowheads* indicate cleaved fragments. *C*, culture supernatant (CS) of LD was immunodepleted using IgG or GP63 antibody, and the immunodepleted supernatant was incubated with J774 cells for 2 h. Then immunoblot was performed using cell lysates and PCBP1 (*upper lane*), PCBP2 (*middle lane*), or actin (*lower lane*) antibody. *D*, immunoriched complex precipitated with IgG or GP63 antibody was incubated with J774 cell lysate for 2 h, and then, immunoblot analysis was performed using PCBP1 (*upper lane*) and PCBP2 (*middle lane*) antibody. Only media after incubation with GP63 antibody were incubated with J774 cell lysate as a control (*first lane*). Quantitation from three independent experiments (±SD) was done and shown in *right panels* for both *C* and *D*. *E*, LD promastigotes were cultured at 37 ^°^C in serum-free RPMI. After 24 h, LD culture supernatant (LD-CS) was concentrated to 25-fold with 30 kDa cutoff filter. Protein in CS was estimated using Bradford reagent, and 500 μg protein was immunodepleted by using IgG or GP63 antibody, and then 60 μg protein was resolved on 10% SDS-PAGE and examined for abundance of GP63 by Western blot analysis (*left panel*). *Middle panel* is the Coomassie staining of the PVDF membrane for verification of loading and transfer. Data represent one of the three independent experiments. Quantitation from three independent experiments (±SD) was done and shown in the *right panel*. IgG, immunoglobulin G; J774, J774A.1; PCBP, poly(rC)-binding protein; PVDF, polyvinylidene difluoride; TPEN, *N*,*N*,*N*′,*N*′-tetrakis (2-pyridinylmethyl)-1,2-ethylenediamine.

**Figure 7:**
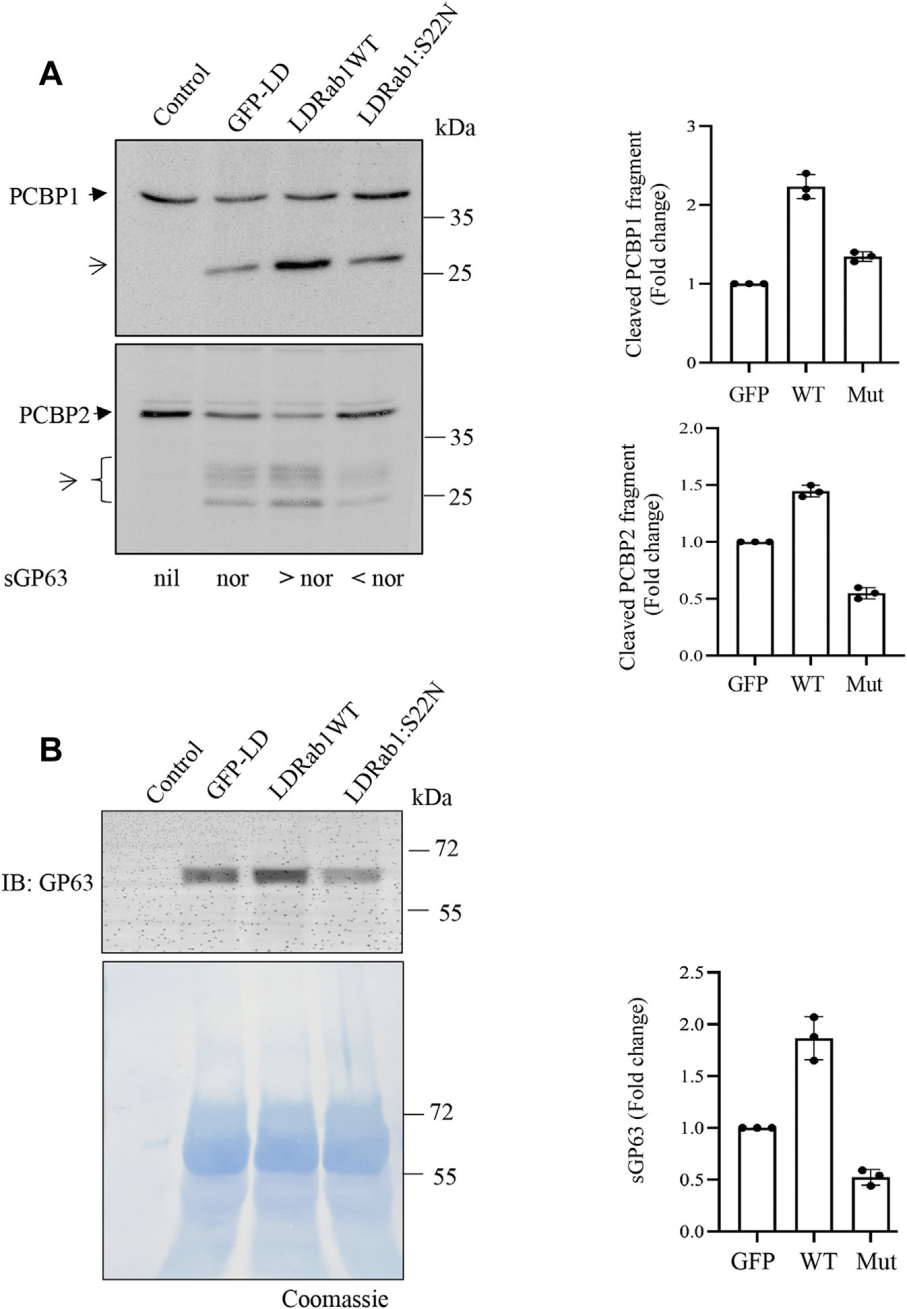
**Effect of culture supernatant (CS) of GP63 secretory mutant of *Leishmania donovani* (LD) on PCBPs.***A*, J774 cells were incubated for 2 h with concentrated CS from transgenic LD overexpressing GFP-LD (transfection control), LDRab1WT, and LDRab1:S22N, and immunoblot analyses were performed for PCBP1, PCBP2, or actin. *Solid arrowheads* indicate intact PCBPs, whereas *open arrowheads* indicate cleaved products. Data shown are representatives of one of the three independent experiments. A relative amount of secretory GP63 (sGP63) compared with normal (nor) has been indicated below the figure. *B*, LD CS of GFP-LD, LDRab1WT, and LDRab1:S22N was collected as described previously and examined for abundance of GP63 by Western blot analysis (*upper panel*). *Lower panel* represents Coomassie-stained blot as loading control. *Right panels* represent quantification (±SD) from three independent experiments. J774, J774A.1; PCBP, poly(rC)-binding protein.

The cleavage of PCBPs in nucleus indicates involvement of a secretory protease of the parasite (Fig. 4) and suggests that the internalization of the parasite may not be essential for LD-induced cleavage of PCBPs. To explore this possibility, prior to infection, J774 cells were treated with cytochalasin D, a known inhibitor of phagocytosis that could block internalization of *Leishmania* (27) and tested for PCBP cleavage. Results showed that cytochalasin D treatment did not block cleavage of PCBP1 and PCBP2 (Fig. 6*A*). Furthermore, J774 cells were incubated with enriched fraction of exosomes derived from LD promastigotes, and cleavages were detected in both PCBP1 and PCBP2 (Fig. 6*B*). The exosome-enriched fraction was independently verified for affecting earlier reported GP63 targets like mammalian target of rapamycin complex 1 or c-Jun (data not shown). Similarly, concentrated culture supernatant (CS) of the parasite also could cleave both PCBP1 and PCBP2 in J774 cells (Fig. S3). These results suggest that a secreted protease from LD cleaves PCBP1 and PCBP2 of the host cells. *Leishmania* is known to cleave multiple host regulatory molecules by secretory GP63, a Zn-containing metalloprotease (27, 28, 29, 30, 31, 32). To verify the possible involvement of GP63, J774 cells were incubated with Zn-chelator TPEN (*N*,*N*,*N*′,*N*′-tetrakis (2-pyridinylmethyl)-1,2-ethylenediamine)-pretreated exosome-enriched fraction, and results showed blocking of cleavages of both PCBP1 and PCBP2 (Fig. 6*B*). Furthermore, J774 cell lysates were incubated with soluble leishmanial antigen (SLA) in the absence or the presence of Zn chelators like *o*-phenanthroline (*o*-phe) or TPEN; a partial reversal of PCBP1 and PCBP2 cleavage was detected (Fig. S4). These results suggest the involvement of a Zn-containing protease in cleavage of PCBPs. Earlier report showed that heat inactivation abrogates GP63 activity (33). Heat-inactivated LD did not cleave either PCBP1 or PCBP2 (Fig. S5). Furthermore, concentrated CS of the parasite was immunodepleted with GP63 antibody prior to incubation with J774 cells. This resulted in significant abrogation of PCBP1 and PCBP2 cleavage (Fig. 6*C*). Incubation of GP63-enriched immunoprecipitated fraction with J774 cell lysates caused cleavages in PCBPs (Fig. 6*D*). The immunodepleted CS was immunoblotted with GP63 antibody to confirm the immunodepletion (Fig. 6*E*). It has recently been reported that Rab1 regulates secretory pathway in LD to control GP63 secretion (34). Overexpression of WT Rab1 (LDRab1WT) and a GDP-locked dominant negative mutant of Rab1 (LDRab1:S22N) could increase and decrease GP63 secretion respectively compared with only vector-transfected parasite (GFP-LD) (34). When we incubated J774 cells with the concentrated CSs derived from these transgenic parasites, differential cleavages were detected in both PCBP1 and PCBP2 compared with CS of only vector-transfected LD (Fig. 7*A*). LDRab1WT that secretes higher GP63 than GFP-LD caused more intense cleavage of both PCBP1 and PCBP2; whereas LDRab1:S22N that secretes less GP63 cleaved less PCBP1 and PCBP2. The difference in secreted amount of GP63 was confirmed by Western blot analysis (Fig. 7*B*) (34). These results strongly suggest the involvement of GP63 in the cleavage of host PCBP1 and PCBP2 by LD.

### LD infection affects Fe loading into Ft in macrophages

**Figure 8:**
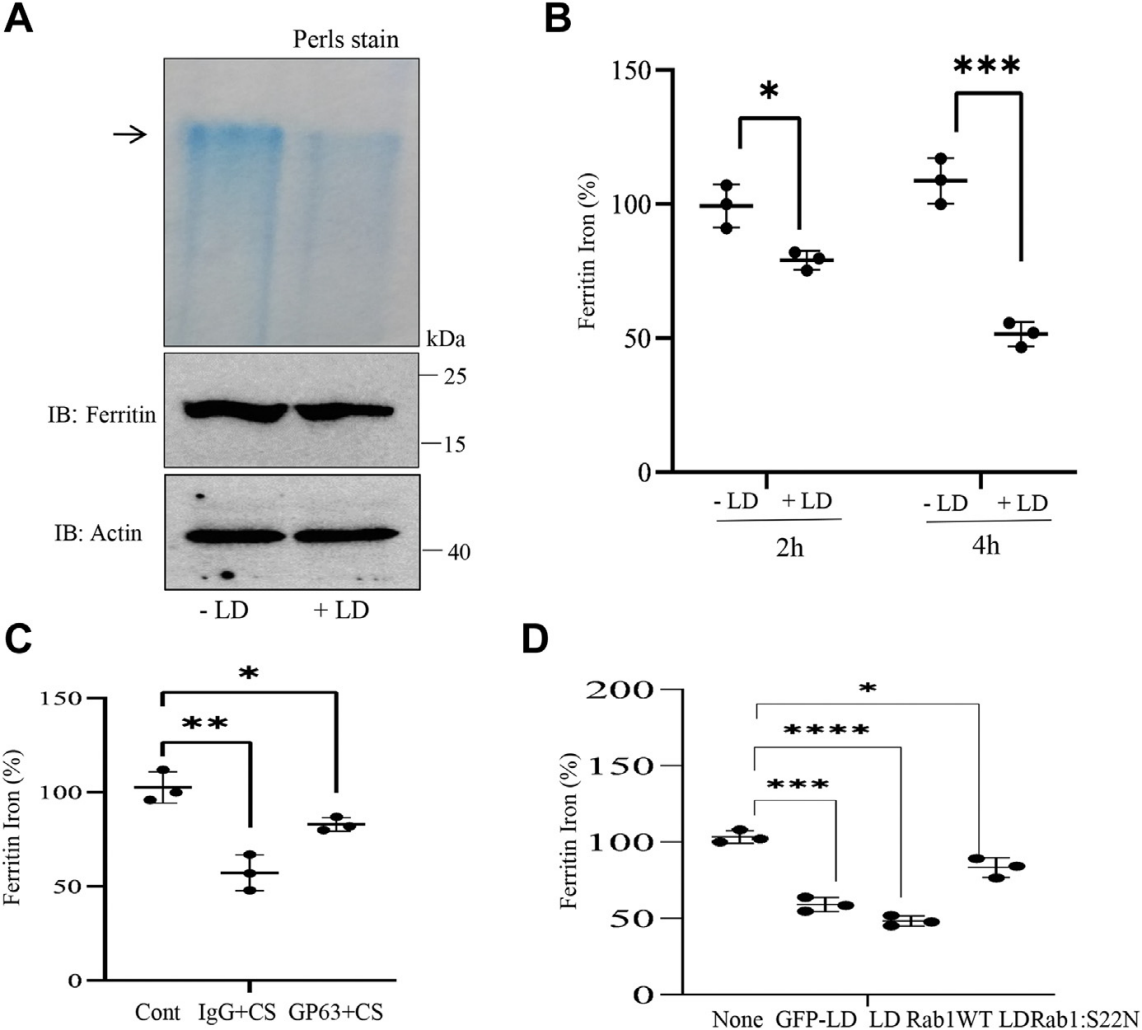
**Estimation of iron (Fe) in ferritin (Ft) in *Leishmania donovani* (LD)-infected macrophages.***A*, cytosolic extracts from LD-infected and LD-uninfected J774 cells (4 h) were subjected to in-gel Perls’ staining. *Arrow* indicates Fe contents in Ft. Western blot analysis for Ft (*middle panel*) and actin (*bottom panel*) was performed from same cytosolic extract. Results represent one of the three independent experiments. *B*, Fe content of Ft was estimated in LD-infected and LD-uninfected splenocytes after 2 and 4 h. ∗*p* < 0.02; ∗∗∗*p* < 0.001. *C*, J774 cells were incubated with concentrated culture supernatant (CS) of LD immunodepleted with GP63 antibody or IgG (4 h), and Ft Fe content was estimated. ∗*p* <0.02; ∗∗*p*< 0.005. *D*, J774 cells were incubated with concentrated CS from GFP-LD (transfection control), LDRab1WT, and LDRab1:S22N transgenic LD for 4 h, and cell lysates were immunoprecipitated with Ft antibody, and Fe content in Ft was estimated by ferrozine assay. ∗∗∗*p* < 0.0005; ∗∗∗∗*p* < 0.0002; ∗*p* < 0.02. Data represent (*B*–*D*) from three independent experiments and represent mean ± SD. Error bars represent ±SD. IgG, immunoglobulin G; J774, J774A.1; PCBP, poly(rC)-binding protein.

We then examined if LD infection in macrophages affects Fe loading into Ft. Cytosolic extracts of infected and uninfected J774 macrophages were passed through a cutoff filter of 100 kDa and subjected to an in-gel assay using Perls’ staining. The Fe content in the >100 kDa cutoff sample (mainly containing Ft as Fe storage component) obtained from infected cells was substantially low in compared with uninfected cells (Fig. 8*A*). The expression of Ft in both samples was verified by Western blot analysis (Fig. 8*A*). To gain a more quantitative view, we further estimated Fe content by ferrozine assay in immunoprecipitated Ft (35). About 30% and 55% decreases in Fe level in Ft were detected in LD-infected splenocytes after 2 and 4 h, respectively, in compared with uninfected cells (Fig. 8*B*). Ft level remained unaltered by infection (Fig. S6). Furthermore, the concentrated CS of the parasite was immunodepleted with either GP63 antibody or immunoglobulin G (IgG) and then incubated with J774 cells. Marginally affected Fe content in Ft was detected with GP63-immunodepleted CS compared with the IgG-incubated CS (Fig. 8*C*). Since CSs of GP63 secretory mutants of LD could influence cleavage of PCBPs, we tested their abilities in loading Fe into Ft. J774 cells were incubated with CSs obtained from vector-transfected parasites or overexpressed Rab1 mutants. CS derived from the GP63 secretory mutant (LDRab1:S22N; secreting less GP63) was less effective in affecting Fe loading into Ft compared with vector-transfected parasites or WT Rab1 (LDRab1WT; secretes higher GP63) (Fig. 8*D*). These results clearly suggest that LD affects Fe loading into Ft in the host cells by cleaving Fe chaperones.

### LD accumulates higher Fe from host for intracellular growth by cleaving PCBPs

**Figure 9:**
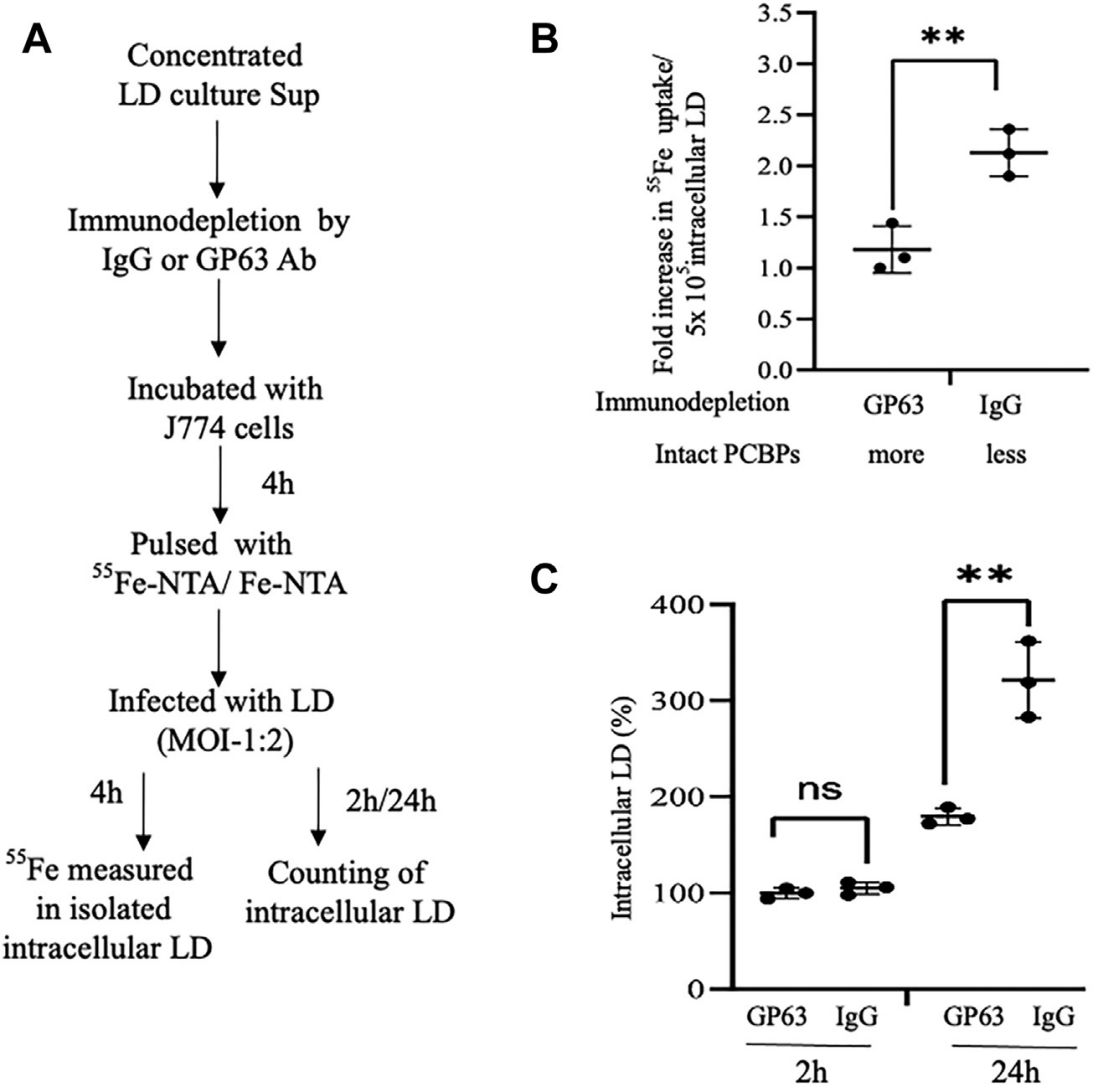
**Cleavage of PCBPs promotes iron (Fe) accumulation and growth of intracellular *Leishmania donovani* (LD).***A*, schematic diagram of experimental design to understand the influence of cleavage of PCBPs on Fe accumulation and growth of intracellular LD. Culture supernatants of LD were concentrated and incubated with either IgG or GP63 antibody. Immunodepleted supernatants were incubated with J774 cells for 4 h, washed, and replenished with the new media. Cells were then pulsed with ^55^Fe–NTA/Fe–NTA for 4 h, washed to remove extracellular radiolabeled/unlabeled Fe, and infected with LD (MOI = 1:2). *B*, after 4 h, intracellular parasites were isolated, and ^55^Fe content was measured. Status of intact PCBPs is mentioned below the figure, ∗∗*p* < 0.006. *C*, intracellular parasites were isolated and counted after 2 and 24 h. Results represent three independent experiments done in triplicates, ∗∗*p* < 0.004. Error bars represent ±SD. IgG, immunoglobulin G; J774, J774A.1; MOI, multiplicity of infection; ns, no significance; NTA, nitrilotriacetic acid; PCBP, poly(rC)-binding protein.

To determine the impact of cleaved PCBPs on the capacity of intracellular *Leishmania* to avail Fe from the host pool; we initially incubated J774 cells with GP63-immunodepleted or IgG-incubated CSs for 4 h to promote differential cleavages of PCBPs as described in earlier experiment (Fig. 6*C*). After changing the media, cells were pulsed with ^55^Fe–nitrilotriacetic acid (NTA) for 4 h. Cells were then washed to remove radiolabeled Fe and infected with LD (MOI = 1:2). Lower MOI was used to minimize the further cleavage of PCBPs. After 4 h of infection, intracellular parasites were isolated and ^55^Fe level was determined (24). Results showed that intracellular *Leishmania* could accumulate about twofold more radiolabeled Fe from the host cells that were incubated with CS treated with control IgG (higher cleavage of PCBPs; Fig. 6*C*) compared with the GP63-immunodepleted CS-treated cells (marginal cleavage of PCBPs; Fig. 6*C*) (Fig. 9*B*). Earlier, we reported the capacity of intracellular LD in using host LIP for its intracellular growth (24). We assumed that the cleavage of Fe chaperones and subsequent higher Fe accumulation from host would help the growth of intracellular parasites. To examine that we adopted a similar strategy (Fig. 9*A*) in which J774 cells were initially incubated with GP63-immunodepleted or IgG-incubated CSs for 4 h and then pulsed with Fe–NTA. After 4 h, media were changed, and cells were infected with *Leishmania* (MOI = 1:2). After 2 h, the numbers of intracellular parasites were found to be similar. However, after 24 h, we detected that about 80% more intracellular *Leishmania* in cells incubated with the control IgG compared with GP63-immunodepleted CSs (Fig. 9*C*). These results imply that intracellular *Leishmania* can avail more Fe from host for its growth by GP63-mediated cleavage of Fe chaperones.

## Discussion

Withholding Fe is a key innate immune strategy of the host to deny availability of this essential micronutrient to the invading pathogens (1, 2). Ft, the principal Fe storage component of the mammalian hosts, plays a crucial role in host–pathogen interaction by storing Fe during infections. PCBP1 and PCBP2 are essential in loading Fe into Ft because of their chaperone activities (6, 10). The current study revealed that intracellular pathogen LD could interfere in Fe loading into Ft in host macrophages by cleaving PCBP1 and PCBP2 using Zn-containing protease GP63 to promote its Fe accumulation and growth within host cells. Our study thus revealed a hitherto unreported novel strategy of a pathogen to interfere with storage capacity of the host cell to acquire Fe for its intracellular growth.

We have provided several evidence of involvement of the leishmanial Zn-metalloprotease GP63 for cleaving PCBPs. We detected partial blocking of cleavage of PCBP1 and PCBP2 using Zn chelators like *o*-phe and TPEN in an *in vitro* system in which the macrophage cell lysates were incubated with SLA. A similar experimental strategy was used earlier to establish the role of GP63 in cleaving Dicer1 (28). GP63 is well known to be secreted from *Leishmania* (27). The blocking of entry of *Leishmania* into host cells by using cytochalasin D did not influence the cleavage of PCBPs suggesting involvement of secretory protease such as GP63. GP63 was earlier reported to be effective in host cytosol and nucleus (27, 32). We found that both PCBP1 and PCBP2 could be cleaved in cytosol and nucleus (Fig. 4). Earlier report suggested that the secretion of GP63 was mediated by exosomes to cleave c-Jun, a component of transcription factor AP1 (27). We also observed that LD-derived exosome-rich fraction could cleave PCBP1 and PCBP2. These cleavages were blocked in the presence of Zn chelator TPEN supporting the involvement of GP63. This metalloprotease is present in different pathogenic *Leishmania* species including LD and *L. major*; whereas nonpathogenic *L. tarentolae* expresses only catalytically inactive GP63 (36). Thus, our observation of host PCBP1–PCBP2 cleavage by LD and *L. major* but not by *L. tarentolae* (Fig. 1) supports the role of GP63. We also found that heat denaturation could affect the protease activity of GP63 (Fig. S5) as reported earlier (33, 37). Furthermore, immunodepletion of GP63 made *Leishmania* CS significantly less effective in cleaving PCBPs (Fig. 6*D*), whereas GP63-enriched immunoprecipitated fraction was highly effective in cleaving PCBPs (Fig. 6*E*). Finally, we detected that CS from GP63 secretion mutant of LD (34) resulted in decreased cleavage of both PCBP1 and PCBP2, whereas increased secretion of GP63 by overexpressed Rab1 resulted in higher cleavage (Fig. 7). All these findings strongly implicated the involvement of GP63 in cleaving PCBPs in host macrophages.

**Figure 10:**
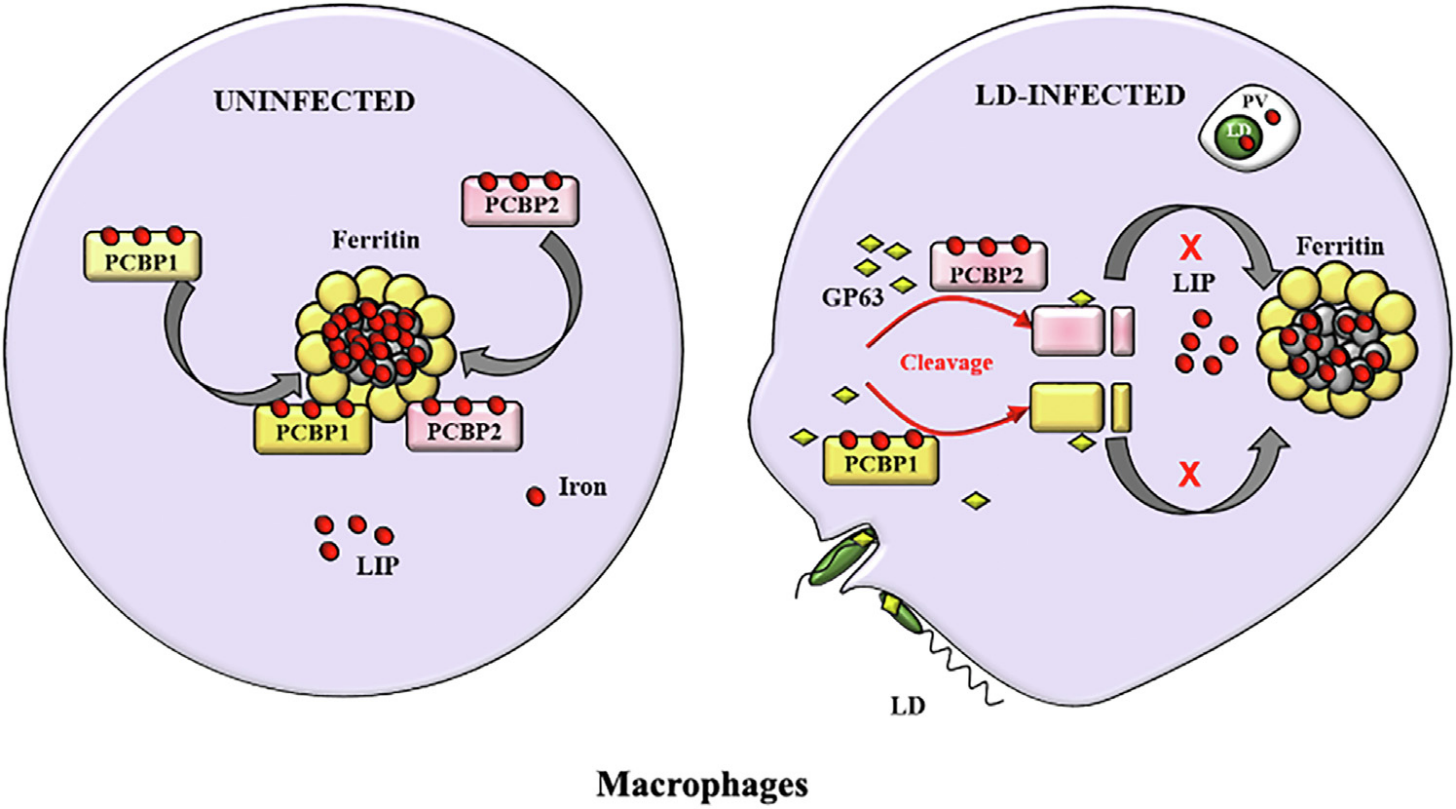
**Schematic representation of mechanism by which *Leishmania donovani* intervenes iron (Fe) loading into host ferritin (Ft).** PCBP1 and PCBP2 are Fe chaperones and bind three atoms of Fe. Both of them load Fe into Ft for storage (*left panel*). During infection of macrophage, the parasite cleaves PCBP1 and PCBP2 using a secreted metalloprotease GP63. Cleaved PCBPs unable to interact with Ft and resulting less Fe loading into Ft. As a consequence, labile iron pool (LIP) of the host becomes more accessible for utilization of intracellular *Leishmania* (*right panel*). PCBP, poly(rC)-binding protein.

GP63, a Zn-dependent metalloprotease, exists abundantly on the surface of LD (33). It is attached on the parasite *via* glycosylphosphatidylinositol anchor. The genes encoding for GP63 exist as a multigene array in the *Leishmania* genome (38). Different gp63 genes have subtle differences in sequence as well as expression pattern (38). However, it is now well established as a critical virulence factor of *Leishmania*, and it interacts with the various host cellular components to modify them for the benefit of the parasite. GP63 was earlier found to impact host cell signaling pathways and transcription factors like AP1 and NF-κB (33). Fe is a critical determinant of the host–parasite interaction (1). Thus, it is advantageous for intracellular *Leishmania* to intervene host Fe sequestration components for accumulation and utilization of host Fe pool for its intracellular growth. So, our finding of GP63-mediated cleavage of Fe chaperones adds on promiscuous activities of this protease. We reported earlier that LD could utilize host LIP to promote its intracellular survival and growth (24). It is well established that Ft can store Fe from LIP (39) and thus may compete with intracellular *Leishmania* for availing Fe from LIP. Thus, it is advantageous for the parasite to cleave chaperones so that storing of Fe into Ft is affected, and intracellular *Leishmania* could avail the Fe pool in a less competitive microenvironment (Fig. 10). In the later stage of infection, *Leishmania* increases Fe uptake by promoting mRNA stability of transferrin receptor 1 (24) and blocking Fe release (25, 40) to increase host cellular Fe pool for its utilization. Taken together, these findings imply that *Leishmania*-induced GP63-mediated cleavage of PCBP1 and PCBP2 may help the parasite, particularly in the early stage of infection.

Storing into Ft is a very well-adopted strategy of the hosts to deny Fe to invading pathogens. The stored Fe becomes almost unavailable to invaders (41). Thus, hosts use different mechanisms to induce Ft to sequester Fe. Infections lead to release of cytokines like interferon-γ and tumor necrosis factor-α, interleukin-1, interleukin-6, and interleukin-10 from monocytes or macrophages and T cells. These cytokines induce Ft expression to stimulate the storage and retention of Fe within macrophages (42). Bacterial surface molecule lipopolysaccharide is also known to induce Ft in various cell types and tissues for enhanced Fe sequestration (3, 43, 44). In this context, the strategy adopted by *Leishmania* to cleave Fe chaperones and to affect Fe loading into Ft is highly favorable to the parasite.

PCBP1 and PCBP2 were also reported to deliver ferrous Fe to enzymes such as prolyl hydroxylases (PHDs) and asparaginyl hydroxylase (FIH1) (45). Together, these enzymes modify the alpha subunit of hypoxia-inducible factor alpha (HIFα), the key regulatory subunit of oxygen-sensing transcription factor HIF1. Earlier, we reported that LD could activate HIF1 in macrophage by mechanisms including HIF1α stabilization for its survival advantage (26). *Leishmania* infection affects PHD activity because of Fe unavailability for HIF1 activation in host cells (26). Our results also may explain HIF1α stabilization because of *Leishmania*-induced cleavage of PCBPs and subsequent decrease in PHD activity in host macrophages. Our study thus implicates the ability of this parasite to adopt to a unique strategy to control Fe and oxygen homeostasis simultaneously by cleaving PCBPs for its survival advantage within host cell.

In summary, our study revealed a novel strategy of the intracellular pathogen *Leishmania* to interfere Fe sequestration into Ft by cleaving Fe chaperones PCBP1 and PCBP2. This helps the parasite to acquire Fe from host for promoting its intracellular growth. PCBPs are involved in many other cellular functions by binding poly(C)-rich regions in DNA and transcripts. We detected cleavage of PCBP1 and PCBP2 in nucleus of infected cells. Thus, it is likely that LD can manipulate other host homeostasis components by cleaving PCBPs to establish and continue its infection. Further studies are needed to understand the total impact of cleavage of PCBPs by *Leishmania* in host cells.

## Experimental procedures

### Parasite culture

LD AG83 (MHOMy∖IN∖1983∖AG83) was cultured in M199 media supplemented with 10% (v/v) heat-inactivated fetal bovine serum (FBS; Cell Clone), 10,000 units/ml penicillin, and 10,000 μg/ml streptomycin (Gibco) at 22 °C as reported earlier (24, 25, 26). Parasite culture was maintained by subculturing at every 4 to 5 days. Virulence of the parasite was maintained by injecting promastigote stage of LD to 4- to 6-week-old female Balb/c mice and then recovered from the spleen of these infected mice as mentioned before (24, 25, 26). *L. major* culture was kindly provided by Dr Chandrima Shaha of National Institute of Immunology and maintained as described earlier (24). *L. tarentolae* was procured from the American Type Culture Collection and cultured in brain heart infusion media (BD Biosciences) supplemented with 10% (v/v) heat-inactivated FBS, 10,000 units/ml penicillin, 10,000 μg/ml streptomycin (Gibco), and hemin (1 mg/100 ml) at 22 °C and maintained by subculturing on every fourth day.

### Generation of LDRab1 and mutants

LDRab1 and its mutants were overexpressed in LD as a GFP fusion protein having GFP tag at the N terminus into the Not1/BamH1 sites of the pXG-GFP2+ vector as described in detail previously (34). Freshly isolated LD promastigotes were transfected with only vector (GFP-LD), LDRab1:WT, or its mutant LDRab1:S22N, and positive clones were selected in the presence of G418 antibiotic (30 μg/ml) as described earlier (34). LDRab1 and relevant mutants were generously provided by Dr Amitabha Mukhopadhyay (National Institute of Immunology).

### Cell culture

Murine macrophage cell J774A.1, RAW264.1, and human monocytic cell lines THP1 and U937 were procured from American Type Culture Collection and maintained in RPMI1640 medium (Sigma–Aldrich) supplemented with 10% (v/v) FBS and 10,000 units/ml penicillin, and 10,000 μg/ml streptomycin in a humidified atmosphere containing 5% CO_2_ at 37 °C in a CO_2_ incubator (24, 25, 26).

### Animals and splenocyte culture

Balb/c mice were used for maintaining virulence of LD as approved by the Institutional Animal Ethics Committee. Four- to 6-week-old mice were used for splenocyte isolation. Female mice were sacrificed, and spleens were taken out and homogenized with the help of frosted glass slides. Isolated splenocytes were pelleted down and cultured in RPMI1640 medium supplemented with 10% (v/v) FBS, 10,000 units/ml penicillin, and 10,000 μg/ml streptomycin in a humidified atmosphere containing 5% CO_2_ at 37 °C in an incubator as described earlier (25).

### Infection of macrophages

Host cells (1 × 10^6^) were seeded in 60 mm culture dish for 24 h and then infected with metacyclic stage of promastigote *Leishmania* at an MOI of 1:10 (host:parasite) if not mentioned otherwise. Parasites were used within 6 weeks after fresh passaging.

### Real-time PCR

RNA from LD-infected and LD-uninfected splenocytes was isolated by using TRIzol (Ambion Life Technologies; catalog no.: 15596026) reagent as per company’s protocol. cDNA was synthesized from 5 μg of purified RNA by using cDNA synthesis kit (Thermo Scientific). Real-time PCR was performed on Bio-Rad CFX96 Real-Time System PCR machine by using SYBR Green (Thermo Scientific; catalog no.: F-416) according to the manufacturer’s protocol. β-Actin was used as endogenous control. Primers to amplify PCBP1 were 5'-CTG CAA GAT CAA GGA GAT CCG-3' (forward) and 5'-AGG CAG ATC TGC TTC ACA CAC-3' (reverse), and for PCBP2 were 5'-CGT CAA GGC GCC AAA ATC AA-3' (forward) and 5'-GCA GCA GAT CCA GTG ATG GT-3' (reverse), and for β-actin were 5'-GAG CGC AAG TAC TCT GTG TG-3' (forward) and 5'-CGC AGC TCA GTA ACA GTC CG-3' (reverse).

### Cloning of mouse PCBP1 cDNA

Total RNA was isolated from J774A.1 cells using TRIzol reagent as mentioned earlier. cDNA was synthesized from the total RNA (4 μg) using MMLV reverse transcriptase enzyme (Epicentre Biotechnologies) as per the manufacturer’s instructions. The mouse PCBP1 cDNA was PCR amplified using specific forward primer consisting of EcoRI restriction site (underlined), 5-'ATA CAT GAA TTC ATG GAC GCC GGT GTG ACT GAA-3' and reverse primer consisting of XhoI restriction site (underlined), 5'-ATA CAT CTC GAG CTA GCT GCA CCC CAT CCC CTT-3'. PCR amplification was performed in 50 μl reaction volume containing 30 pM each of forward and reverse primer, 0.03 mM deoxy NTPs, 0.1 mM MgSO_4_, and five units of precision Taq polymerase (Applied Biological Material, Inc). The reaction was set at conditions as follows: 94 °C for 5 min, 25 cycles of 94 ^°^C for 30 s, 55 ^°^C for 30 s, and 72 ^°^C for 1.5 min. Final extension was carried out for 7 min at 72 ^°^C. Amplified product was purified, double digested, and cloned into pcDNA3 vector between EcoRI and XhoI sites. The clone was verified by sequencing.

### Cloning of mouse PCBP1 cDNA into p3xFLAG-CMV-7.1 expression vector

The coding region of mouse PCBP1 gene was PCR amplified using pcDNA3-PCBP1 plasmid as template and subcloned into HindIII and SmaI restriction sites of p3xFLAG-CMV-7.1 expression vector. Following primers were used for the PCR amplification: forward: 5'-ATA CAT AAG CTT ATG GAC GCC GGT GTG ACT-3' and reverse: 5'-ATA CAT CCC GGG CTA GCT GCA CCC CAT CCC-3'. The clone was verified by sequencing.

### Cloning of mouse PCBP1 cDNA into p3xFLAG-CMV-14 expression vector

PCBP1 coding sequence was PCR amplified from the pcDNA3-PCBP1 construct and subcloned into HindIII and KpnI restriction sites of p3xFLAG-CMV-14 expression vector. For the amplification and expression of the PCBP1 protein, forward primer was designed to incorporate a kozak sequence (AACC) before the initiation codon ATG, and the reverse primer was without stop codon to put the PCBP1 coding region in frame with 3xFLAG sequence. Following primers were used: forward, 5'-ATA CAT AAG CTT AAC CAT GGA CGC CGG TGT GAC TGA A-3' and reverse, 5'-ATA CTA GGT ACC GTT GCG CTG CAC CCC ATC CCC TTC TCA GA-3'. The clone was confirmed by sequencing.

### Cloning of mouse PCBP2 variants into p3XFLAG-CMV-7.1 expression vector

PCBP2 coding region was PCR amplified from cDNA synthesized as mentioned earlier using forward primer 5'-TAC GTA AGC TTA TGG ACA CCG GTG TGA TTG AAG-3' and reverse primer 5'-TCA GTC CCG GGC TAG CTG CTC CCC ATG CCA CC-3'. Two amplicons were detected, purified, digested with restriction enzymes HindIII and SmaI, and separately cloned into p3xFlag-CMV-7.1 vector. Both of them were verified by sequencing and identified as PCBP2 variant 1 and variant 3 (National Center for Biotechnology Information no.: NM_001103165.1 and NM_001103166, respectively).

### Western blot analysis

Cells were washed twice with chilled 1× PBS and then lysed in ice-cold lysis buffer (50 mM Hepes [pH 7.5], 150 mM NaCl, 1 mM EDTA, 1 mM PMSF, 2 mM sodium vanadate, 0.5% NP-40 [v/v], and 1× protease inhibitor cocktail [Roche Diagnostics]). Protein was estimated by Bradford reagent (Bio-Rad), and 40 μg protein was separated on SDS-PAGE (12%) and then transferred on to polyvinylidene difluoride membrane (Millipore). Membranes were blocked with 5% nonfat skimmed milk and incubated overnight with PCBP1 (1:5000 dilution; Abcam, catalog no.: ab74793), PCBP2 (1:5000 dilution; Novus Biologicals, catalog no.: NBP1-57323), Ft (1:5000 dilution; Novus Biologicals, catalog no.: NB 600-920), M2 monoclonal antibody (1:5000 dilution; Sigma–Aldrich, catalog no.: F1804), actin (C-11; 1:2000 dilution; Santa Cruz Biotechnology, catalog no.: sc-1615), GAPDH (v-18; 1:1000 dilution, Santa Cruz Biotechnology, catalog no.: sc 20357), lamin A (H-102; 1:1000 dilution; Santa Cruz Biotechnology, catalog no.: sc-20680), and hnRNP K (1:1000 dilution; Santa Cruz Biotechnology, catalog no.: sc-28380) antibodies. After incubation with primary antibody, membranes were washed with 1× Tris-buffered saline with Tween-20 three times and then incubated in antimouse (1:5000 dilution; Sigma–Aldrich, catalog no.: A4416), anti-rabbit (1:5000 dilution; Pierce, catalog no.: 31460) and antigoat (1:5000 dilution; Santa Cruz Biotechnology, catalog no.: sc-2020) secondary antibodies conjugated with horseradish peroxidase and then developed with enhanced chemiluminescence (ECL kit; Amersham). GP63 secreted by transgenic parasites was determined by Western blot analysis as described earlier (34).

### Isolation of nuclear and cytosolic fractions

Cells were grown in 100 mm dishes. After 24 h, cells were transfected with either N-terminal-tagged PCBP1-FLAG or PCBP2-FLAG variant 1 or 3 construct by Lipofectamine 2000 (Invitrogen). On third day of seeding, cells were infected with *Leishmania*. After 2 h of infection, cells were washed and scraped in ice-cold 1× PBS. Nuclear fraction was isolated as described earlier (24, 46). In short, cells were first washed with 1 ml buffer A (25 mM Hepes, pH 7.5, 5 mM KCl, 0.5 mM MgCl_2_, 1 mM DTT, 40 μM PMSF, aprotinin 10 μg/ml, and leupeptin 10 μg/ml) and then suspended in buffer A + NP-40 (0.5%) (v/v) on ice. After 5 min, intact nuclei were settled down by centrifugation at 600*g* for 2 min. Nuclei were again washed with buffer A + NP-40 (0.5%) (v/v). Then washing was done with buffer B (25 mM Hepes [pH 7.5], 10% glycerol, 0.01% NP-40 [v/v], 40 μM PMSF, 1 mM DTT, aprotinin 10 μg/ml, and leupeptin 10 μg/ml). Nuclei were resuspended in 40 μl buffer B + NaCl (350 mM) for 1 h. Nuclear lysate was centrifuged at 10,000*g* for 15 min. Supernatant was taken and resolved in SDS-PAGE (12%) and subjected to Western blotting. For cytosolic extract preparation, cells were suspended in buffer containing 50 mM Tris–Cl (pH 7.6), 50 mM NaCl, 1 mM DTT, 1 mM PMSF, and 1× protease inhibitor cocktail. Cell suspension was subjected to three rounds of freeze–thaw cycles followed by passing through 26-gauze needle for four to five times and was centrifuged at 15,000*g* for 30 min at 4 ^°^C (25). Supernatants containing cytosolic fractions was preserved at −80 ^°^C for further use.

### Co-IP

J774 cells were transiently transfected with p3xFLAG-CMV-7.1-PCBP1 (PCBP1-FLAG) and p3xFLAG-CMV-7.1-PCBP2-variant 1 (PCBP2V1-FLAG) plasmids using Lipofectamine 2000 and then infected with LD (2 h). Cell lysate was prepared by using lysis buffer (50 mM Tris–Cl, 150 mM NaCl, 0.5% NP-40 [v/v], 1 mM PMSF, 1 mM DTT, and EDTA-free protease inhibitor cocktail [Sigma–Aldrich]). Cell lysates (1.5 mg protein) were immunoprecipitated using M2 beads (Sigma–Aldrich; catalog no.: A2220) or mouse IgG (Santa Cruz Biotechnology; catalog no.: sc-2025) and resolved in 15% SDS-PAGE and immunoblotted with Ft or FLAG antibody. For reverse IP, PCBP1-FLAG– and PCBP2-FLAG–transfected cells were infected with parasite. After 2 h, cell lysates were prepared using aforementioned buffer, and cell lysate (3 mg protein) was subjected to IP with anti-Ft antibody (Novus Biologicals; catalog no.: NB 600-920) or rabbit IgG (Invitrogen; catalog no.: 02-6192) and A/G beads (Santa Cruz Biotechnology; catalog no.: sc-2003), and then, complexes were resolved using 15% SDS-PAGE. Interacting partners were analyzed by Western blot analysis by using anti-Ft antibody (1:5000 dilution; Abcam; catalog no.: ab7332) and anti-FLAG antibody (1:5000 dilution; Sigma–Aldrich).

### Preparation of concentrated LD CS and exosome-enriched supernatant

LD promastigotes were cultured at 37 ^°^C in serum-free RPMI. After 24 h, *Leishmania* CS and same volume of RPMI were concentrated to 25-fold using 30 kDa cutoff filter (Millipore) and used as concentrated CS. An earlier study reported significant increase in the release of exosome by *Leishmania* at higher temperature (37 ^°^C) (47). So, after 24 h of incubation at 37 ^°^C, LD promastigote culture was pelleted at 900*g* for 10 min, and supernatant was filtered twice with 0.22 micron filter (Millipore). Then supernatant was concentrated with 30 kDa cutoff filter, and concentrated conditioned media were washed with 1× PBS and ultracentrifuged at 100,000*g* for 70 min to pellet down exosomes as described earlier (48). It was again washed with 1× PBS and ultracentrifuged at 100,000*g* for 70 min and used as exosome-enriched supernatant. In some cases, exosome-enriched fraction was incubated with TPEN (10 μM) or *o*-phe (10 μM) for 30 min in ice.

### *In vitro* PCBP1 and PCBP2 cleavage assay

*In vitro* PCBP1 and PCBP2 cleavage assay was performed as described previously (28). SLAs were prepared as described earlier with minor modifications (49). For SLA preparation, parasite was lysed in buffer containing 10 mM Tris–HCl (pH 7.5), 1 mM DTT, 100 mM KCl, and 1× protease inhibitor cocktail by three freeze–thaw cycles and then followed by sonication (three pulses of 15 s of 25% amplitude with interval of 2 min). The suspension was centrifuged at 6000*g* for 20 min, and the supernatant containing leishmanial antigens was used for assay. J774 cell extract prepared in aforementioned buffer was coincubated with SLA for 30 min. To chelate Zn, SLA was pretreated with either *o*-phe (10 μM) or TPEN (10 μM) for 30 min at 4 ^°^C. Samples were subjected to Western blot analysis using PCBP1, PCBP2, or actin antibody.

### Immunodepletion of GP63 in concentrated LD CS

Concentrated CS of the parasite containing 500 μg protein was subjected to immunoprecipitation using mouse anti-GP63 antibody (LifeSpan BioSciences, Inc; catalog no.: LS-C58984), mouse IgG (Santa Cruz Biotechnology; catalog no.: sc-2025), and A/G beads at 4 ^°^C overnight. Supernatant was collected separately in a tube and preserved, and immunoprecipitated samples were washed thrice with IP buffer (50 mM Tris–Cl, 150 mM NaCl, 0.5× protease inhibitor cocktail, and 0.5 mM DTT). Then, immunoprecipitated samples were coincubated with J774 cell lysates for an hour. A concentrated media control was also included and coincubated with J774 cell lysates. Furthermore, samples were separated on 12% SDS-PAGE and subjected to immunoblot analysis using anti-PCBP1, anti-PCBP2, and antiactin antibody. The immunodepleted supernatant was collected separately in a tube and incubated with J774 cells for determining the effect on PCBP1 or PCBP2.

### Estimation of Fe in Ft

#### In-gel assay

J774 cells were infected with LD (4 h, MOI = 1:10) or kept uninfected, and then, cytosolic fractions were prepared as described earlier. Cytosolic fractions containing Ft of ∼500 kDa size were passed through 100 kDa cutoff filters (Millipore). The concentrated cytosolic fraction of >100 kDa was then loaded in 3 to 8% polyacrylamide gel under native condition. The gel was then subjected to Perls’ blue staining to detect the incorporated Fe^3+^ presumably in the Ft. Perls’ staining was performed by submerging the gel in 0.1% potassium ferrocyanide in 0.1 N HCl for 45 min to 2 h, and then, stained gels were washed several times with 0.1 N HCl and stored in the dark in 0.1 N HCl until photographed.

#### Ferrozine assay

Fe content in Ft in J774 cells and splenocytes were estimated as described earlier (35). Ft was immunoprecipitated from uninfected and infected cells after differential treatments as described in the respective figure legends. After IP, Ft was eluted into 50 μl SDS sample buffer with β-mercaptoethanol without bromophenol blue at 95 ^°^C for 5 min. The Ft protein abundance was verified by Western blot analysis. The eluate (50 μl) was added with concentrated HCl (11 μl, 11.6 M) and incubated at 95 ^°^C for 20 min. After centrifugation for 10 min at 12,000*g*, 45 μl of supernatant was added to 18 μl ascorbic acid (75 mM) for reducing the Fe. Ferrozine (18 μl, 10 mM) was added, and the reaction was stopped with 36 μl saturated ammonium acetate (NH_4_OAc). Absorbance of the samples was measured at 562 nm and compared with a standard curve. Ft Fe content in uninfected cell was taken as 100% to compare with infected cells.

### Preparation of radiolabeled Fe and loading into J774 cells

A solution of ^55^Fe–NTA was prepared by mixing ^55^FeCl_3_ (PerkinElmer) with a fivefold molar excess of disodium salt of NTA (50). J774 cells (5 × 10^6^) were incubated with ^55^Fe–NTA (1 μM) in serum-free medium for 4 h. Cells were washed twice with 150 mM NaCl containing 10 μM EDTA to remove surface-bound Fe.

### Isolation and measurement of radiolabeled Fe in intracellular parasite

J774 cells were incubated with GP63/IgG-immunodepleted CSs for 4 h and pulsed with ^55^Fe–NTA for another 4 h. Then cells were washed to remove excess radiolabeled Fe and infected with LD. After 30 min, media were changed to remove extracellular parasites. Intracellular parasites were isolated after 4 h and counted using percoll gradient as described earlier (24, 25, 26, 51). In short, infected macrophages were lysed using four freeze–thaw cycles. Cell lysates were then subjected to percoll gradient (in the order of 90, 40, and 20%) and spun at 800*g* for 1 h. The band from the interface of 90/40% percoll was collected, and the volume was equilibrated up to 1 ml. Then parasites were counted in the improved Neubauer Counting Chamber, and ^55^Fe were detected in isolated LD in a scintillation counter (24). The number of intracellular parasites was also counted similarly after 2 and 24 h of infection as described earlier (24, 25, 26, 51).

### Statistical analysis

All experiments were performed at least three times with similar results, and representative experiments are shown. Data are expressed as the mean ± SD. All statistical tests were calculated using GraphPad Prism, version 8.4.2 (679) software. A *p* value <0.05 was used to indicate significance.

## Data availability

All data are contained within the article.

## Supporting information

This article contains supporting information

## Conflict of interest

The authors declare that they have no conflicts of interest with the contents of this article.
