# Supplementary material for: Intracellular pathogen Leishmania intervenes in iron loading into ferritin by cleaving chaperones in host macrophages as an iron acquisition strategy
Source: J Biol Chem. 2022 Oct 26;298(12):102646. doi: 10.1016/j.jbc.2022.102646 (PMC9700016; doi:10.1016/j.jbc.2022.102646)
Supplement: Supplemental Figures S1–S6 [file mmc1.pdf]

Supporting Information

Intracellular pathogen *Leishmania* intervenes in iron loading into ferritin by cleaving chaperones in host macrophages as an iron acquisition strategy

*Sandhya Sen, *[$]Saswat Kumar Bal, Sameeksha Yadav, Pragya Mishra, Vishnu Vivek G, Ruchir Rastogi, and [#]Chinmay K. Mukhopadhyay

From the Special Centre for Molecular Medicine, Jawaharlal Nehru University, New Delhi-110067, India

Running title: *Leishmania* cleaves host iron chaperones

*Contributed equally; [$]Present address: SS: Department of Gastroenterology and Hepatology, Mayo Clinic, Minnesota 55905; SKB: Department of Pathology, Emory University, Atlanta, GA 30322

List of the materials:

S-1: Effect of *L. donovani* (LD) infection on PCBPs in J774 cells.

S-2: Effect of *L. donovani* infection on the expressions of PCBP transcripts.

S-3: *L. donovani* culture supernatant cleaves PCBP1 and PCBP2 in J774 cells.

S-4: Effect of Zinc chelation on Soluble Leishmanial Antigen-induced cleavage of PCBPs in J774 cell lysates.

S-5: Effect of heat inactivated *L. donovani* on PCBP1 and PCBP2 in J774 cells.

S-6: Effect of *L. donovani* infection on ferritin protein expression in mouse splenocytes.

S-1

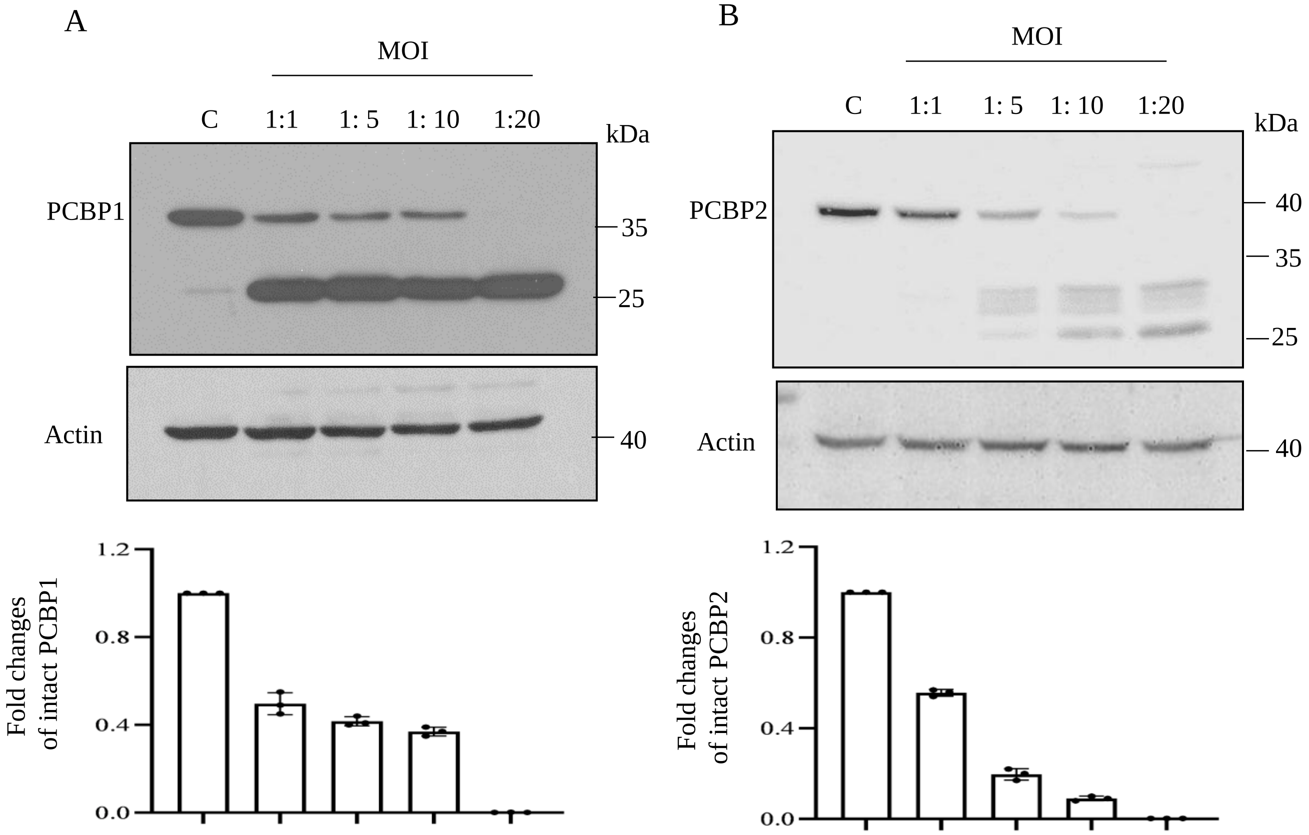

S-1: Effect of *L. donovani* (LD) infection on PCBPs in J774 cells. J774 macrophages were kept uninfected (C) or infected with increasing MOI (1:1 to 1: 20; J774: LD) of parasite for 2h and immunoblot analyses were performed using PCBP1 (A) and PCBP2 (B) antibodies. Actin was used as a loading control. Lower panels represent quantitation from three independent experiments.

S-2

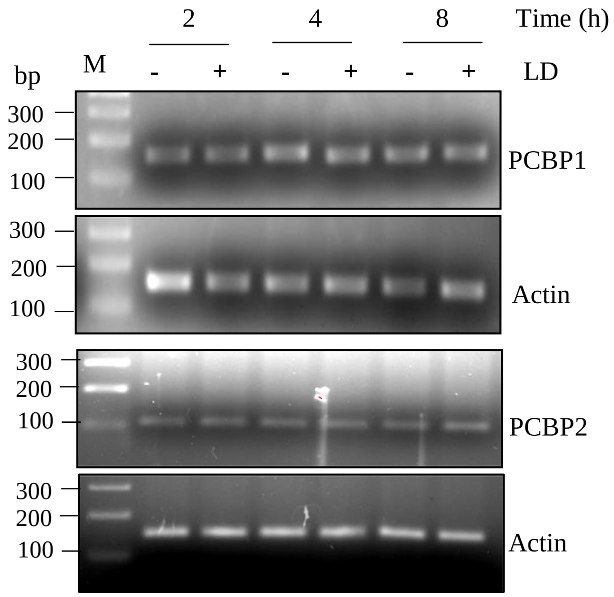

S-2: Effect of *L. donovani* infection on the expressions of PCBP transcripts. J774 macrophages were kept uninfected or infected with *L. donovani* (MOI of 1:10: J774: LD) and total RNA was isolated. PCBP1, PCBP2 and actin mRNA expressions were determined using semi-quantitative RTPCR. The data represent one of the three independent experiments with similar results.

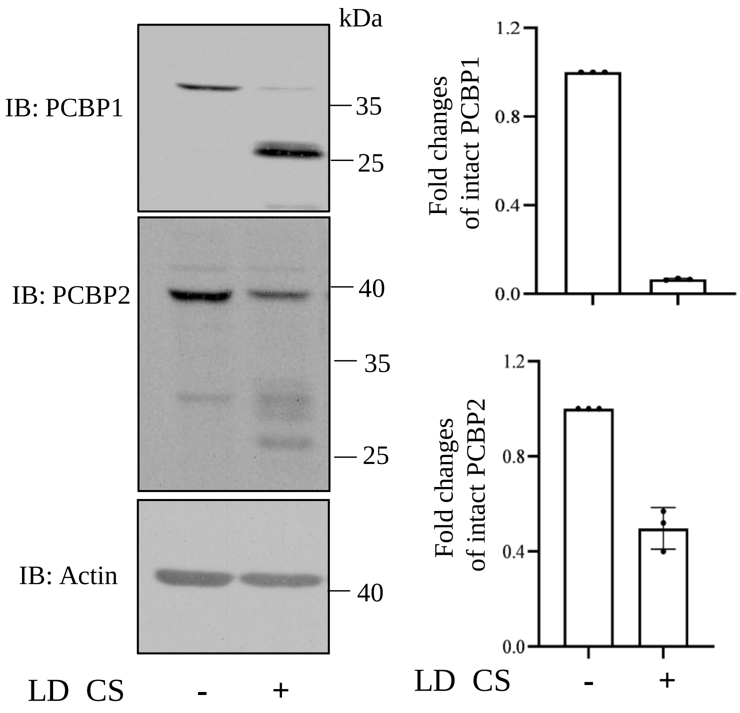

S-3 : *L. donovani* (LD) culture supernatant cleaves PCBP1 and PCBP2 in J774 cells. Promastigotes stage parasites were cultured at 37^{0}C in serum free RPMI. After 24h, conditioned media (CS) and similar volume of RPMI were concentrated to 25 fold using 30 kDa cut off filter (Millipore) and then co-incubated with J774 cells for 2h. Cell lysates were subjected to immunoblot analysis using PCBP1, PCBP2 and Actin antibodies. Right panel represents quantitation from three independent experiments.

S-4

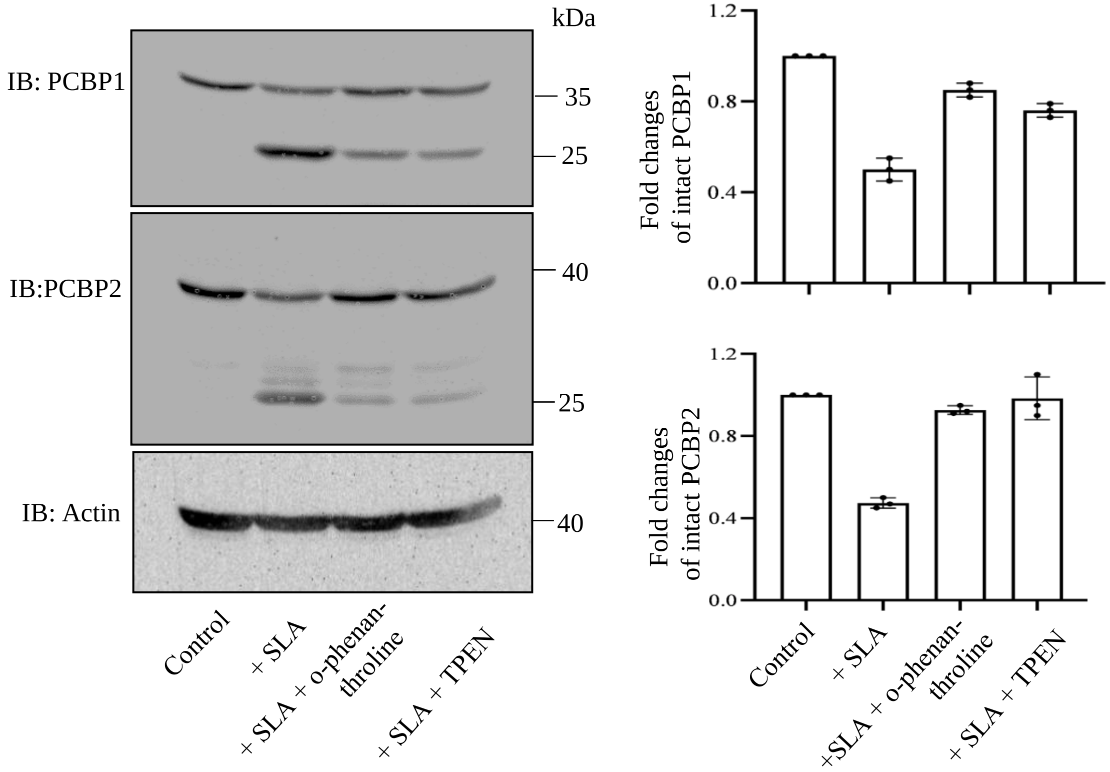

S-4: Effect of Zinc chelation on Soluble Leishmanial Antigen-induced cleavage of PCBPs in J774 cell lysates. Soluble Leishmanial Antigen (SLA) was added with zinc chelators o-phenanthroline (o-phen, 10µM) and TPEN (10µM) or kept alone for 30 min at 4^0C. Then, J774 cell extracts were incubated with SLA $\pm$ zinc chelators mixtures for 30 min at 37^0C and were immunoblotted for PCBP1 and PCBP2. Actin was used as loading control. Right panels represent quantitation from three independent experiments.

S-5

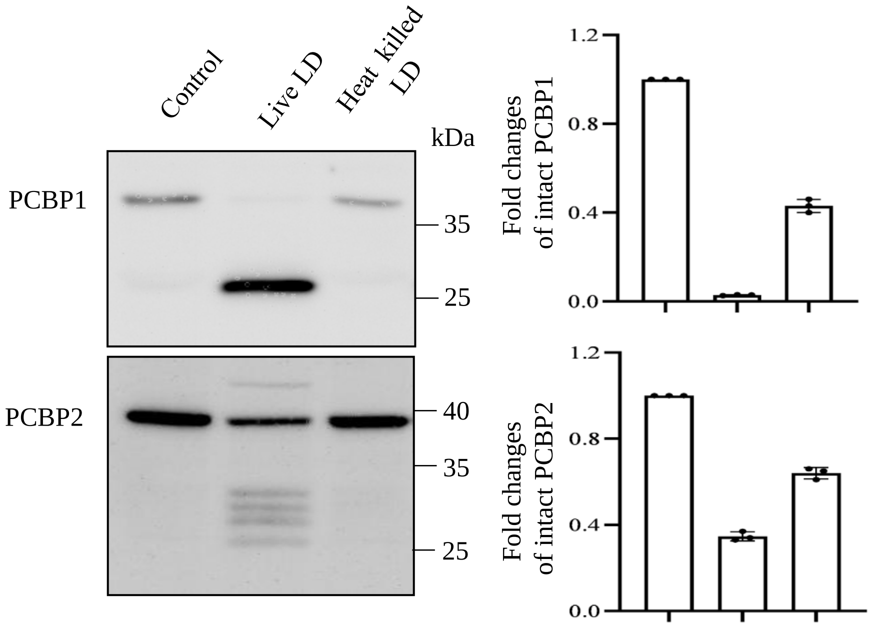

S-5: Effect of heat inactivated *L. donovani* on PCBP1 and PCBP2 in J774 cells. LD was heat inactivated by incubating at 65^{0}C for 20 min. J774 cells were kept uninfected (Control) and infected/incubated with live (MOI-1:10)/heat killed LD for 2h and subjected to immunoblot analysis using PCBP1, PCBP2. Right panels represent quantitation from three independent experiments.

S-6

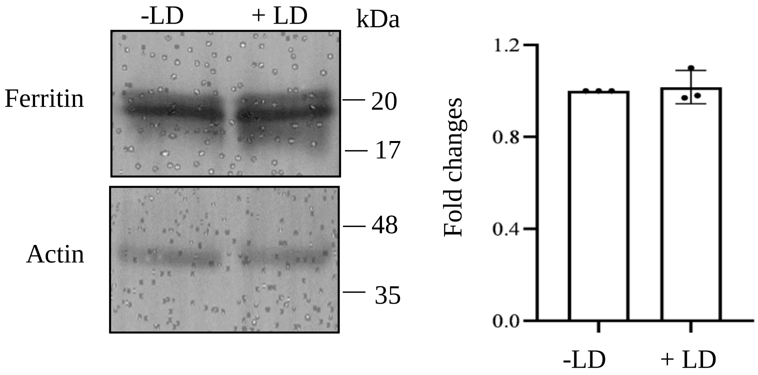

S-6 : Effect of *L. donovani* infection on ferritin protein expression in mouse splenocytes. Total ferritin level was determined in LD-infected and uninfected (MOI- 1:10; 4h) splenocytes isolated from mouse by Western Blot analysis. Actin was used as a loading control. Right panel represents quantitation from three independent experiments.
